# The Plasmodium falciparum Nuclear Protein Phosphatase NIF4 Is Required for Efficient Merozoite Invasion and Regulates Artemisinin Sensitivity

**DOI:** 10.1128/mbio.01897-22

**Published:** 2022-08-08

**Authors:** Xiaotong Zhu, Siqi Li, Chengqi Wang, Yuanchao Yu, Jingjing Wang, Lu He, Faiza Amber Siddiqui, Lumeng Chen, Liying Zhu, Dan Zhou, Junling Qin, Jun Miao, Liwang Cui, Yaming Cao

**Affiliations:** a Department of Immunology, College of Basic Medical Science, China Medical University, Shenyang, Liaoning, China; b Department of Internal Medicine, Morsani College of Medicine, University of South Florida, Tampa, Florida, USA; NIAID/NIH

**Keywords:** malaria, protein phosphatase, invasion, RNA polymerase II, asexual development

## Abstract

Artemisinin resistance in Plasmodium falciparum has been associated with a mutation in the NLI-interacting factor-like phosphatase PfNIF4, in addition to the mutations in the Kelch13 protein as the major determinant. We found that PfNIF4 was predominantly expressed at the schizont stage and localized in the nuclei of the parasite. To elucidate the functions of PfNIF4 in P. falciparum, we performed PfNIF4 knockdown (KD) using the inducible ribozyme system. PfNIF4 KD attenuated merozoite invasion and affected gametocytogenesis. PfNIF4 KD parasites also showed significantly increased *in vitro* susceptibility to artemisinins. Transcriptomic and proteomic analysis revealed that PfNIF4 KD led to the downregulation of gene categories involved in invasion and artemisinin resistance (e.g., mitochondrial function, membrane, and Kelch13 interactome) at the trophozoite and/or schizont stage. Consistent with PfNIF4 being a protein phosphatase, PfNIF4 KD resulted in an overall upregulation of the phosphoproteome of infected erythrocytes. Quantitative phosphoproteomic profiling identified a set of PfNIF4-regulated phosphoproteins with functional similarity to FCP1 substrates, particularly proteins involved in chromatin organization and transcriptional regulation. Specifically, we observed increased phosphorylation of Ser2/5 of the tandem repeats in the C-terminal domain (CTD) of RNA polymerase II (RNAPII) upon PfNIF4 KD. Furthermore, using the TurboID-based proteomic approach, we identified that PfNIF4 interacted with the RNAPII components, AP2-domain transcription factors, and chromatin-modifiers and binders. These findings suggest that PfNIF4 may act as the RNAPII CTD phosphatase, regulating the expression of general and parasite-specific cellular pathways during the blood-stage development.

## INTRODUCTION

Malaria is a significant global public health burden, causing 409,000 deaths in 2019 (1). Plasmodium falciparum is the deadliest of the five human *Plasmodium* species. With the rising resistance of P. falciparum to artemisinin derivatives, the first-line antimalarial drugs, new therapies are urgently needed. Although the P. falciparum life cycle entails several morphologically distinct developmental stages, only the cyclic asexual multiplication in the red blood cells (RBCs) is associated with the disease symptoms (2). During the intraerythrocytic developmental cycle (IDC), merozoites invade RBCs and progress from newly formed ring-stage parasites to mature schizonts, while each schizont releases 16 to 24 progeny merozoites to infect new RBCs. Erythrocyte invasion is critical for parasite survival and proliferation, and this process is regulated by signal transduction mediated by reversible protein phosphorylation catalyzed by protein kinases and phosphatases (3–5). While protein kinases have long been recognized as important drug targets, protein phosphatases have only recently received attention as potential therapeutic targets.

*Plasmodium* genomes encode approximately 30 predicted protein phosphatases (PPs), divided into four major groups, including the PPP (phospho-protein phosphatases) group, the PPM/PP2C group, the PTP (protein tyrosine phosphatase) superfamily, and the NIF (NLI interacting factor-like phosphatase) group (6). Gene disruption analysis in Plasmodium berghei has revealed that 53% of these predicted PPs are essential for asexual development, and six are required for development in the mosquito (7). Several studies have characterized the molecular functions of PPs in development. For example, PP1 is implicated in playing a role in merozoite egress, PP5 is responsible for male gamete fertilization, PPM2 is essential for gametocytogenesis and the subsequent ookinete differentiation, PPM5 is required for oocyst development in the mosquito, and PTPLA is required for sporozoite development (7–10). However, the functions of the NIF family phosphatases remain enigmatic.

In mammalian cells and yeast, the NIF group includes the Fcp1 (TFIIF-associated C-terminal domain [CTD] phosphatase 1) and Scp (Small CTD phosphatases) (11, 12). The Fcp/Scp family members rely on the DxDxT/V motif to dephosphorylate the CTD of RNA polymerase II (RNAPII), which contains tandem repeats of a serine-rich heptapeptide Y^1^S^2^P^3^T^4^S^5^P^6^S^7^. Both the level and pattern of the CTD YSPTSPS motif phosphorylation oscillate with the transcription cycles, with hypophosphorylation in the preinitiation complex and hyperphosphorylation during transcription elongation (13–15). In P. falciparum, the NIF group includes NIF2 (PF3D7_0515900), NIF3 (PF3D7_1355700), NIF4 (PF3D7_1012700), and TIM50 (6). Except for TIM50, all three NIF members share a DxDx(T/V) signature motif and are essential for asexual development (7). In P. falciparum, RNAPII acts as a general transcription factor regulating the transcription of mRNAs and most small nuclear RNAs (snRNAs). Notably, the CTD heptapeptide motif of the PfRNAPII subunit Rpb1 is slightly different from the conventional motif and possesses a lysine instead of serine at position 7 (Y^1^S^2^P^3^T^4^S^5^P^6^K^7^). Like in other eukaryotes, phosphorylation of Ser5 of the YSPTSPK motif in P. falciparum is required for RNAPII to initiate transcription, while di-phosphorylation of Ser2 and 5 confers the elongating and highly processive activity of RNAPII (16). Similarly, it is also possible that dephosphorylation of the YSPTSPK motif is required to recycle PfRNAPII to reinitiate the transcription cycle. Although conserved proline-directed kinases, including a yeast Ctk1 kinase homolog (PF3D7_0417800) and a human CDK7 homolog (PF3D7_1014400), were predicted to be responsible for YSPTSPK phosphorylation in P. falciparum (16), it remains to be tested whether the NIF phosphatases carry out the function of RNAPII CTD dephosphorylation in this parasite.

The emergence and spread of artemisinin (ART)-resistant P. falciparum parasites in the Greater Mekong subregion (GMS) have raised global concerns for malaria control (17, 18). ART resistance is manifested as delayed parasite clearance, with mutations in the propeller domain of the Kelch13 (K13) gene as the major determinant (19). Population transcriptomic analysis revealed substantial transcriptome changes in resistant parasites and altered IDC with decelerated development at the young ring stage (20). In addition to K13 mutations, genome-wide association studies (GWAS) have identified additional genomic loci strongly associated with clinical ART resistance, including *ferredoxin*, *apicoplast ribosomal protein S10*, *multidrug resistance protein 2*, *chloroquine resistance transporter* (*crt*), and the protein phosphatase *pfnif4* (21). In addition, PfNIF4 mutations were also associated with reduced *in vitro* susceptibility of the parasites to dihydroartemisinin (DHA) and artemether (22). Although these genomic loci are considered background alleles on which ART resistance arose, it is also possible that they play supplementary roles in ART resistance. These findings have prompted us to investigate the function of PfNIF4 in transcriptional regulation and its potential involvement in ART resistance. We studied the function of PfNIF4 during the IDC using the *glmS* self-cleaving ribozyme inducible knockdown (KD) system and found that PfNIF4 KD significantly perturbed erythrocyte invasion by the merozoites and increased the parasite’s susceptibility to ARTs. In agreement with these phenotypes, transcriptomic and proteomic analysis revealed that PfNIF4 KD resulted in the downregulation of multiple invasion-related genes and dysregulation of several pathways implicated in ART resistance. Furthermore, we used a proximity-based proteomic approach to identify interactions between PfNIF4 with RNAPII subunits and transcriptional regulators. Phosphoproteome analysis revealed upregulated phosphorylation on sites of the Rpb1 CTD heptad repeats, GCN5, and several major epigenetic factors in PfNIF4 KD parasites. Together with the increased phosphorylation of RNAPII CTD motifs upon PfNIF4 KD, we corroborated the function of PfNIF4 as a consensus Fcp/Scp phosphatase in P. falciparum.

## RESULTS

### PfNIF4 is expressed during both asexual and sexual development.

The functional significance of NIF family PPs in regulating RNAPII activity and transcription prompted us to study the functions of PfNIF4 in P. falciparum. PfNIF4 belongs to the Fcp1 family of NIF-like phosphatases (7). It contains an N-terminal CPDc (catalytic domain of ctd-like phosphatases) domain (322 to 675 amino acids [aa]) but lacks the BRCT domain (breast cancer protein-related carboxy-terminal) that is found in the yeast Fcp1 protein (Fig. S1A). PfNIF4 also contains several Lys-rich motifs that were predicted as nuclear localization signals. The PfNIF4 CPDc domain shows >60% aa similarity to those of Fcp1 proteins in model organisms. It also contains the Fcp1 signature motif DxDx(T/V), which is known to bind metal during catalysis (23) (Fig. S1B). A NIF4 ortholog is present in all *Plasmodium* species with the same Fcp1 signature motif DLDNT, suggesting a conserved catalytic mechanism of this protein in malaria parasites (Fig. S1C). Structure prediction using I-TASSER showed a conserved ctd-like phosphatase fold of the PfNIF4 catalytic domain, which aligned well with the catalytic domain of FCP1 from Schizosaccharomyces pombe (Fig. S1D and E).

10.1128/mbio.01897-22.1 FIG S1 Bioinformatics of NIF4 protein in P. falciparum. Download FIG S1, PDF file, 1.9 MB. Copyright © 2022 Zhu et al. 2022 Zhu et al. https://creativecommons.org/licenses/by/4.0/ This content is distributed under the terms of the Creative Commons Attribution 4.0 International license.

**FIG 1:**
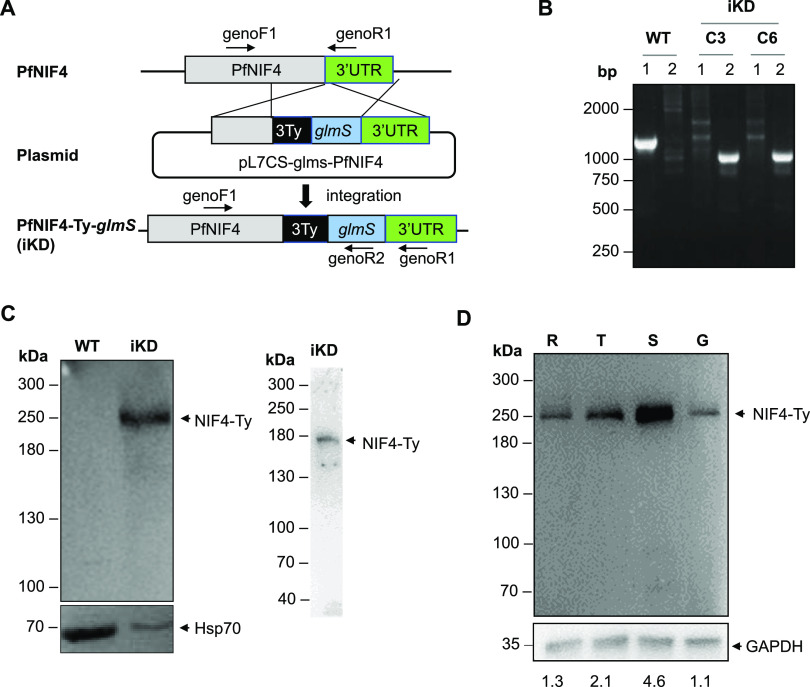
Expression of PfNIF4-Ty protein during asexual and gametocyte development. (A) Diagram showing the integration of the Ty-ribozyme sequence at the *pfnif4* locus using the CRISPR/cas9 method. The positions of primers (genoF1, genoR1, and genoR2) are indicated. (B) Confirmation of Ty-*glmS* integration at the *pfnif4* locus by integration-specific PCR using genomic DNA from NIF4^iKD^ clones C3 and C6 (iKD) and the parental 3D7 strain (WT). Lane 1, primers genoF1+genoR1 (WT, 1,243 bp; iKD,1,628 bp); Lane 2, primers genoF1+genoR2 (iKD, 982 bp). (C) Western blot analysis of WT and NIF4^iKD^ parasite lysates of asynchronous cultures with (right panel) or without (left panel) alkaline phosphatase treatment using the anti-Ty antibody. Arrows indicate the position of the NIF4-Ty protein. Protein loading per lane was verified using the anti-Hsp70 MAb. (D) Western blot of PfNIF4-Ty protein expression at the ring (R), trophozoite (T), schizont (S), and gametocyte (G) stages of NIF4^iKD^ parasites with the anti-Ty antibody. GAPDH was used as the protein loading control. The relative NIF4-Ty/GAPDH signal intensity ratios calculated with the Image J software are shown below the gel image.

To study PfNIF4 expression, we generated a transgenic parasite (NIF4^iKD^) with the PfNIF4 C-terminus fused to a triple-Ty tag followed by the *glmS* ribozyme (24) using the CRISPR/Cas9 genome engineering method (Fig. 1A). After transfection and drug selection, the NIF4^iKD^ clones were isolated via limiting dilution. Diagnostic PCR confirmed the correct integration of the Ty tag and ribozyme at the *pfnif4* locus in the tested parasite clones C3 and C6 (Fig. 1B). Western blot analysis with the anti-Ty monoclonal antibody (MAb) confirmed the expression of the tagged PfNIF4-Ty protein in mixed asexual stage parasites. The Ty-tagged PfNIF4 migrated at ~250 kDa, larger than the predicted molecular size of PfNIF4 (174.5 kDa). Because previous phosphoproteome analysis identified several phosphorylation sites in PfNIF4 (4, 5, 25), we speculated that the slower migration of PfNIF4 in the SDS-PAGE gel might be due to phosphorylation. To test this possibility, we pretreated the parasite protein lysate with alkaline phosphatase before gel separation. Phosphatase treatment resulted in PfNIF4-Ty migrating at around 175 kDa, indicating the occurrence of phosphorylation on PfNIF4 (Fig. 1C).

Transcriptomic studies showed that *pfnif4* was expressed in all developmental stages with increased transcript levels in schizonts (www.plasmodb.org). To study the dynamics of PfNIF4 protein expression during development, we performed Western blot analysis with the anti-Ty MAb using lysates of highly synchronized rings, trophozoites, schizonts, and purified stage V gametocytes from the NIF4^iKD^ parasites. The ~250 kDa PfNIF4-Ty protein band was detected in all the stages tested, with the peak protein level observed at the schizont stage, consistent with the transcription profile of *pfnif4* (Fig. 1D).

**FIG 2:**
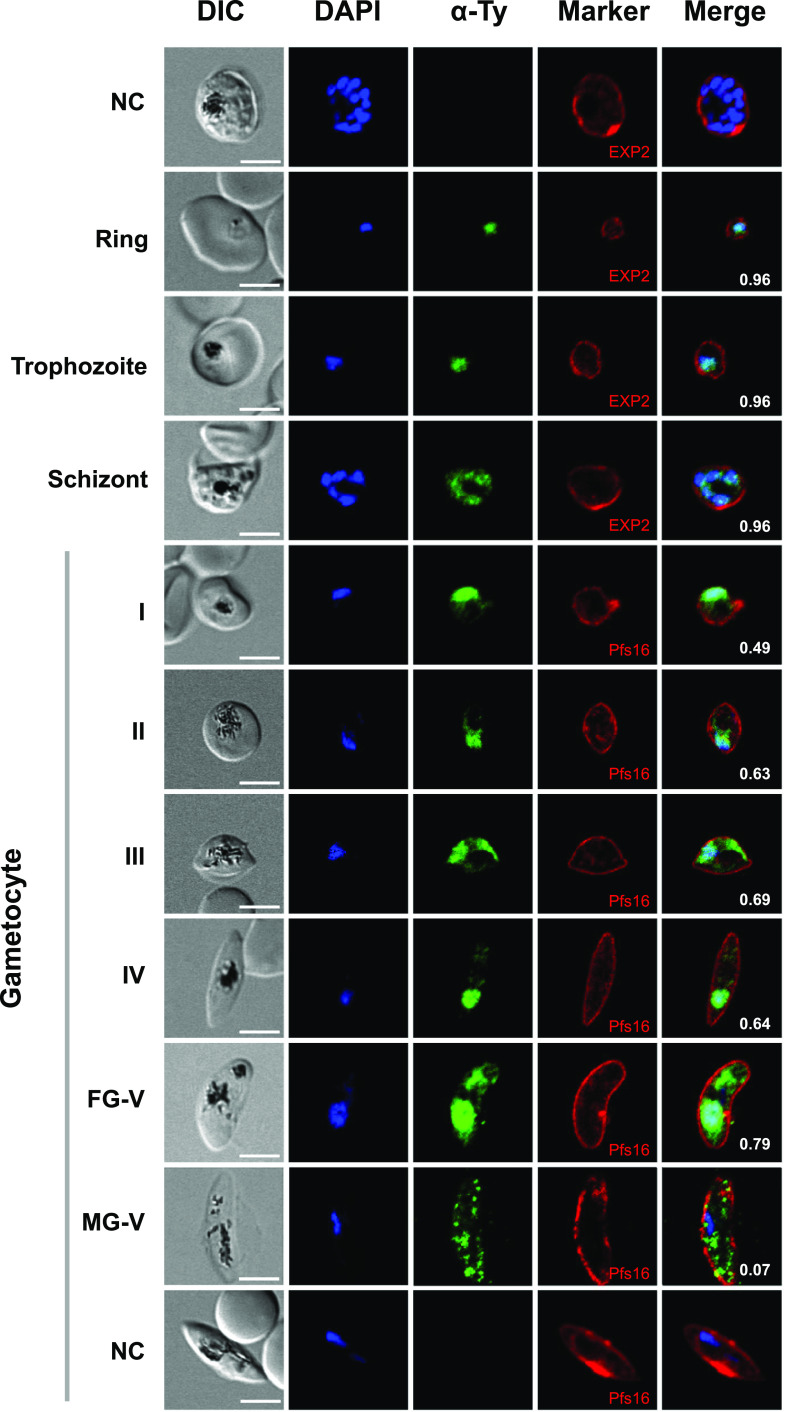
Subcellular localization of PfNIF4-Ty protein during asexual and gametocyte stages by IFA. Representative images of the ring, trophozoite, schizont, and stage I to V gametocytes showing localization of the PfNIF4-Ty protein. Parasites were probed with the anti-Ty MAb (mouse, 1:500) together with anti-EXP2 sera (rabbit, 1:500, for asexual stage parasites) or anti-Pfs16 (rabbit, 1:500, for gametocytes). Secondary antibodies were anti-mouse Alexa Fluor 488 (green) and anti-rabbit Alexa Fluor 594 (red). The IFA analysis of schizonts and gametocytes of the parental 3D7 strain (WT) stained with anti-EXP2 sera and anti-Pfs16 antibody were used as negative control (NC). The nuclei were visualized with DAPI (blue). Numbers in the merged images show the Pearson’s correlation coefficients for DAPI/Ty in the NIF4^iKD^ strain. Scale bar: 5 μm. DIC, differential interference contrast image; FG, female gametocyte; MG, male gametocyte.

Indirect immunofluorescence assays (IFA) were performed to investigate the subcellular localization of PfNIF4-Ty in asexual-stage parasites and gametocytes. During the IDC, PfNIF4-Ty was localized within the parasitophorous vacuole membrane (PVM) defined by the PVM protein EXP2 (Fig. 2). As expected for a potential role in transcriptional regulation, the PfNIF4-Ty signal was observed primarily in the parasite nucleus with relatively even distribution and high colocalization with the nuclear staining by DAPI (Pearson’s correlation coefficient [PCC], 0.96) (Fig. 2). During gametocyte development, PfNIF4-Ty was detected in all gametocyte stages and both male and female gametocytes (Fig. 2). Unlike in asexual stages, PfNIF4-Ty signals in stage I to IV gametocytes showed a partial colocalization with the nuclear marker (PCC, 0.49 to 0.66), and there was an uneven distribution of the fluorescence in the cytoplasm (Fig. 2). IFA in mature female gametocytes further confirmed a partial colocalization of PfNIF4-Ty with the nuclear marker (PCC, 0.79) (Fig. 2), whereas PfNIF4-Ty was detected throughout the cytoplasm in mature male gametocytes, with little or no overlap with the DAPI signal (PCC, 0.07) (Fig. 2).

### PfNIF4 KD affects parasite proliferation.

**FIG 3:**
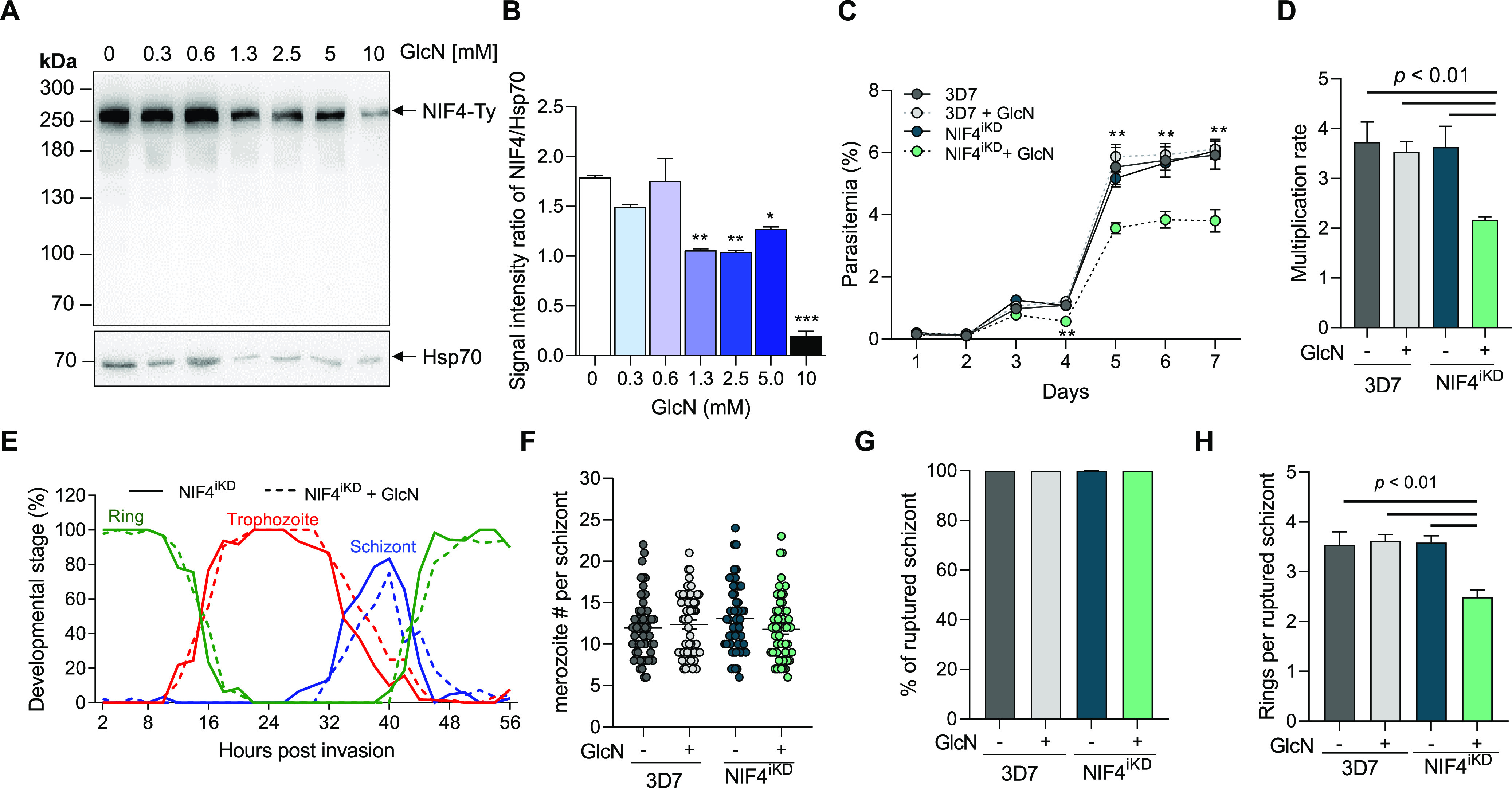
Phenotypic characterization of PfNIF4 KD across the asexual life cycle. (A) Western blot analysis of NIF4-Ty protein expression in the presence of 0, 0.3, 0.6, 1.3, 2.5, 5, and 10 mM GlcN. Protein loading per lane was monitored by the anti-Hsp70 MAb. (B) Analysis of relative NIF4-Ty protein KD in panel A by using the Image J software. ** (*P* < 0.01) and *** (*P* < 0.001) indicate statistical comparisons of PfNID4 band intensity between the control and GlcN-treated parasites. (C) Asexual growth of the 3D7 and NIF4^iKD^ (clone C3) parasites cultured in the presence or absence of 2.5 mM GlcN. All parasites were seeded at 0.1% parasitemia and 2% hematocrit and parasitemia was monitored daily using Giemsa-stained smears. ** indicates a significant difference in parasitemia between the GlcN-treated NIF4^iKD^ parasites and untreated parasites. (D) Multiplication rates of the parasites. The multiplication rate was determined for each clone as the fold increase in parasitemia per 48 h measured over three cycles. (E) Comparison of the progression of the ring, trophozoite and schizont stage across the IDC using the synchronized NIF4^iKD^ C3 clone in the presence or absence of 2.5 mM GlcN. At each time point, the percentages of the ring (green), trophozoite (red) and schizont (blue) stages are shown. The cultures were monitored every 2 h through a 56-h period. (F) Merozoites number/schizont. The number of merozoites in each mature schizont was counted under a light microscope. (G) The percentages of ruptured schizonts in the 3D7 and NIF4^iKD^ parasites cultured in the presence or absence of 2.5 mM GlcN. (H) Comparison of the merozoite invasion efficiency. The number of rings per ruptured schizont was determined for each parasite line under the specified GlcN treatment conditions.

Next, we wanted to investigate if PfNIF4 KD affected parasite development using the NIF4^iKD^ line. We first titrated the effect of glucosamine (GlcN) concentrations on PfNIF4-Ty protein levels in the NIF4^iKD^ parasite. Synchronized ring-stage parasites at 3 h postinvasion (hpi) were treated with 0, 0.3, 0.6, 1.3, 2.5, or 5 mM GlcN for 32 h, and the PfNIF4 expression levels were assessed by Western analysis. Using the anti-Ty MAb, we found that treatment with 1.3 and 2.5 mM GlcN significantly reduced the PfNIF4-Ty level compared to no GlcN treatment in the NIF4^iKD^ parasites (*P* < 0.01, Fig. 3A and B). Additionally, 2.5 mM GlcN treatment did not affect the asexual growth or gametocytogenesis of the 3D7 parasite (Fig. S2A and B). These results showed that we could achieve a substantial KD of PfNIF4 at GlcN concentrations that caused no noticeable impact on the wild-type (WT) 3D7 parasite.

10.1128/mbio.01897-22.2 FIG S2 The effect of 2.5 mM GlcN on wild-type 3D7 (A, B) and NIF4^iKD^ (C, D) parasites. Download FIG S2, PDF file, 0.10 MB. Copyright © 2022 Zhu et al. 2022 Zhu et al. https://creativecommons.org/licenses/by/4.0/ This content is distributed under the terms of the Creative Commons Attribution 4.0 International license.

To study the effect of PfNIF4 KD on parasite growth, we treated the 3D7 and NIF4^iKD^ parasites with 2.5 mM GlcN starting at 3 hpi (0.1% parasitemia) and followed the daily parasitemia for a week. Although there was no significant difference in parasitemia during the first IDC, PfNIF4 KD impaired parasite growth in the subsequent cycles (Fig. 3C). For example, in GlcN-treated NIF4^iKD^ parasites, parasitemia reached 3.5% on day 5 compared with >5% in untreated NIF4^iKD^ and 3D7. Consistent with the lack of effect of 2.5 mM GlcN on the control parasites, the parental 3D7 line multiplied at a rate of ~3.6 with or without GlcN treatment. In contrast, the PfNIF4 KD parasites displayed a significantly lower multiplication rate of 2.2 (*P < *0.01) (Fig. 3D). To determine whether PfNIF4 KD affected the progress of the IDC, we followed the dynamics of different stages of synchronized parasite cultures at a 2-h interval. We did not observe apparent differences in the progression of individual stages between GlcN-treated and untreated NIF4^iKD^ parasites (Fig. 3E). In addition, PfNIF4 KD did not significantly affect schizogony because GlcN-treated parasites produced similar numbers of merozoites per schizont as the untreated parasites and 3D7 (*P = *0.97, Fig. 3F). Further, the proportion of ruptured schizonts was similar between the treated and untreated parasites, indicating that PfNIF4 KD did not affect the merozoite egress (Fig. 3G). However, GlcN treatment reduced the number of newly formed rings per ruptured schizont to 2.5 compared with 3.6 in untreated parasites, corresponding to a 30.5% reduction in invasion (Fig. 3H). These results altogether indicate that PfNIF4 KD impaired parasite growth by reducing the capacity of the merozoites to invade RBC.

Because PfNIF4 was expressed throughout gametocyte development and showed a sex-specific distribution in mature gametocytes, we investigated whether PfNIF4 KD influenced gametocytogenesis. When NIF4^iKD^ parasites were induced to undergo gametocytogenesis, GlcN-treated NIF4^iKD^ parasites produced similar numbers of mature gametocytes with no apparent morphological abnormality compared to untreated parasites, suggesting that PfNIF4 is not essential for gametocytogenesis (Fig. S2C). Meanwhile, PfNIF4 KD did not significantly affect the male/female ratio, despite the drastic difference in PfNIF4 distribution in male and female gametocytes (Fig. S2D).

### PfNIF4 KD alters the susceptibility of the parasites to artemisinin drugs.

**FIG 4:**
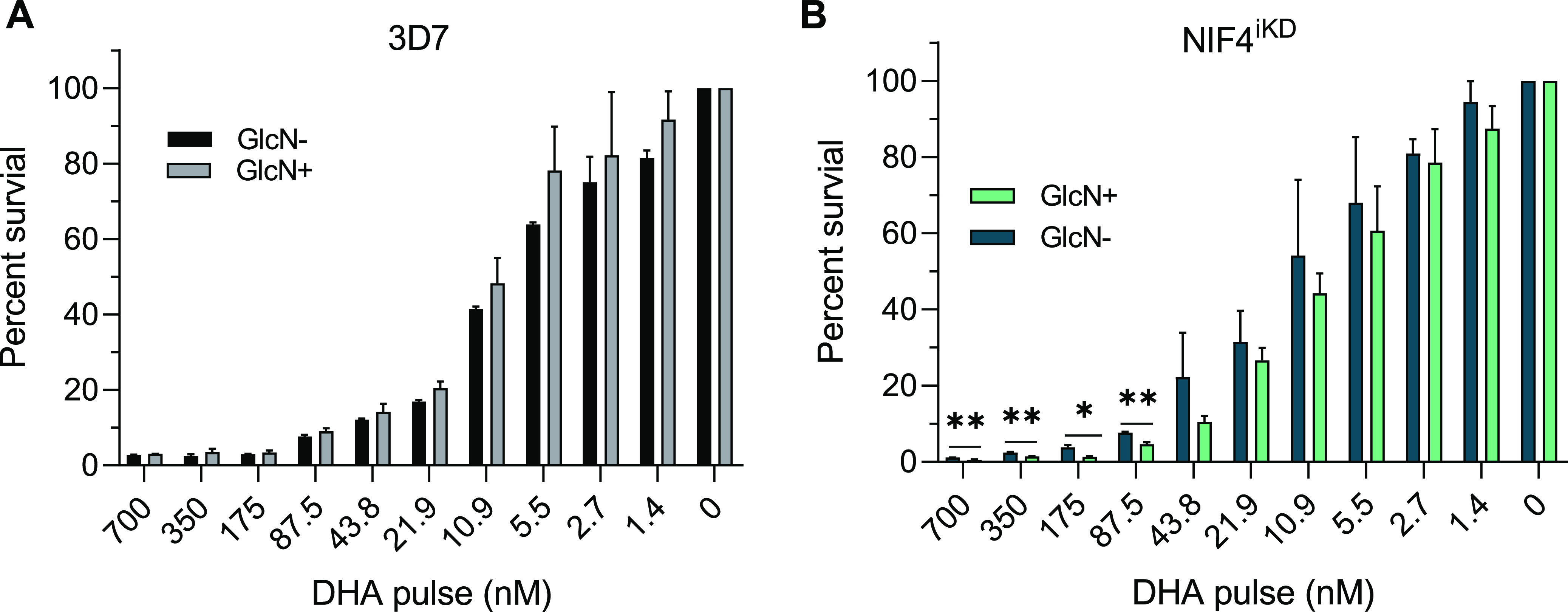
Ring-stage survival assays of the 3D7 and NIF4^iKD^ parasites. Tightly synchronized 3D7 (A) and NIF4^iKD^ (B) parasites at the early ring (0 to 3 hpi) stage were exposed to a 3-h pulse of DHA at the indicated concentrations. Parasite viability was determined by Giemsa-stained smear at 72 hpi. Bar graphs correspond to the percent survival (mean + SD), equivalent to the parasitemia of DHA-treated parasites divided by the parasitemia of the DMSO-treated parasites. Data are from three or four independent experiments, each conducted in duplicate. Statistical significance between GlcN-treated and -untreated parasites was determined by two-tailed Student’s *t* tests. *, *P* < 0.05; **, *P* < 0.01; ***, *P* < 0.001.

To corroborate the involvement of PfNIF4 in ART resistance (21), we evaluated whether the PfNIF4 protein level affected the parasite’s susceptibility to ART using the ring-stage survival assay (RSA) and the standard proliferation-based drug assay. The same assays were conducted in parallel using the 3D7 and NIF4^iKD^ parasites with or without 2.5 mM GlcN treatment. For RSA, tightly synchronized ring stage (0 to 3 h) parasites were exposed to a 6-h pulse of 700 nM DHA *in vitro*, and parasite survival was measured 66 h later (26). In the control 3D7 parasite, 2.5 mM GlcN treatment did not alter the RSA_0–3h_ values when RSA was performed using a gradient of DHA concentrations ranging from 1.4 to 700 nM (Fig. 4A). Using the NIF4^iKD^ parasite line, we found that the RSA_0–3h_ value for a 6-h pulse of 700 nM DHA in the GlcN+ parasites (0.03% ± 0.02%) was significantly lower than that for the GlcN– parasites (0.07% ± 0.04%). Similarly, when the NIF4^iKD^ parasites were exposed to the DHA concentration gradient, the GlcN+ parasites had significantly lower RSA_0–3h_ values than the GlcN– parasites at the DHA concentration of 700, 350, 175, and 87.5 nM (Fig. 4B), indicating PfNIF4 KD increased the DHA susceptibility of ring-stage parasites.

We further expanded the drug susceptibility analysis to a panel of 10 antimalarial drugs and compared the 50% inhibitory concentration (IC_50_) values between 3D7 and NIF4^iKD^ using the traditional SYBR green I-based drug assay (27). The results showed that the presence of 2.5 mM GlcN did not affect the susceptibilities of the 3D7 parasite to any of the drugs tested (Table 1). However, treatment with 2.5 mM GlcN significantly reduced the IC_50_ values of the NIF4^iKD^ parasites to artemether and DHA (*P* < 0.05, Mann-Whitney *U* test), whereas the susceptibilities of the NIF4^iKD^ parasites to other drugs such as the quinolines (chloroquine, piperaquine, amodiaquine) and aminoalcohols (mefloquine, lumefantrine, quinine) were not significantly altered (Table 1). These results collectively indicate that PfNIF4 KD increased the parasite’s susceptibility to ART family drugs.

**TABLE 1:** *In vitro* susceptibilities (IC_50_ in nM) of the WT and NIF4^iKD^ parasites to 10 antimalarial drugs^*a*^

Parasite	AMQ^*e*^	AS^*f*^	AM^*g*^	DHA^*h*^	CQ^*i*^	MFQ^*j*^	NQ^*k*^	LMF^*l*^	PPQ^*m*^	QN^*n*^
3D7–^*b*^	7.3 ± 2.2	3.4 ± 0.2	1.3 ± 0.0	0.6 ± 0.3	49.6 ± 5.7	23.4 ± 6.6	12.9 ± 3.8	23.9 ± 2.6	20.8 ± 5.7	41.4 ± 2.9
3D7+	6.5 ± 3.2	3.5 ± 0.1	1.4 ± 0.4	0.6 ± 0.0	51.2 ± 8.3	24.2 ± 1.2	16.7 ± 1.8	21.4 ± 0.3	16.6 ± 3.1	40.8 ± 6.2
iKD–	5.3 ± 0.1	3.8 ± 0.6	1.5 ± 0.1	0.8 ± 0.0	45.2 ± 3.5	17.8 ± 1.9	14.3 ± 4.5	25.0 ± 3.0	25.4 ± 6.1	40.2 ± 6.9
iKD+	5.2 ± 0.2	3.3 ± 0.0	0.8 ± 0.1^*c*^	0.5 ± 0.0^*d*^	41.2 ± 9.7	18.3 ± 3.0	11.3 ± 0.8	25.6 ± 5.2	24.5 ± 3.7	41.9 ± 2.2

a The IC_50_ values were measured using the 72-h SYBR green I-based assay. Values represent mean ± SD from three independent experiments performed in triplicates. + and – indicate 3D7 and NIF4^iKD^ parasites assayed in the presence or absence of 2.5 mM GlcN, respectively.

b Statistical significance was determined between pairs of + and – by the two-tailed Mann-Whitney *U* test.

c *P* < 0.05.

d *P* < 0.01.

e AMQ, amodiaquine dihydrochloride dihydrate.

f AS, artesunate.

g AM, artemether.

h DHA, dihydroartemisinin.

i CQ, chloroquine diphosphate.

j MFQ, mefloquine hydrochloride.

k NQ, naphthoquine.

l LMF, lumefantrine.

m PPQ, piperaquine.

n QN, quinine.

### PfNIF4 KD disturbs genome-wide transcription during the IDC.

**FIG 5:**
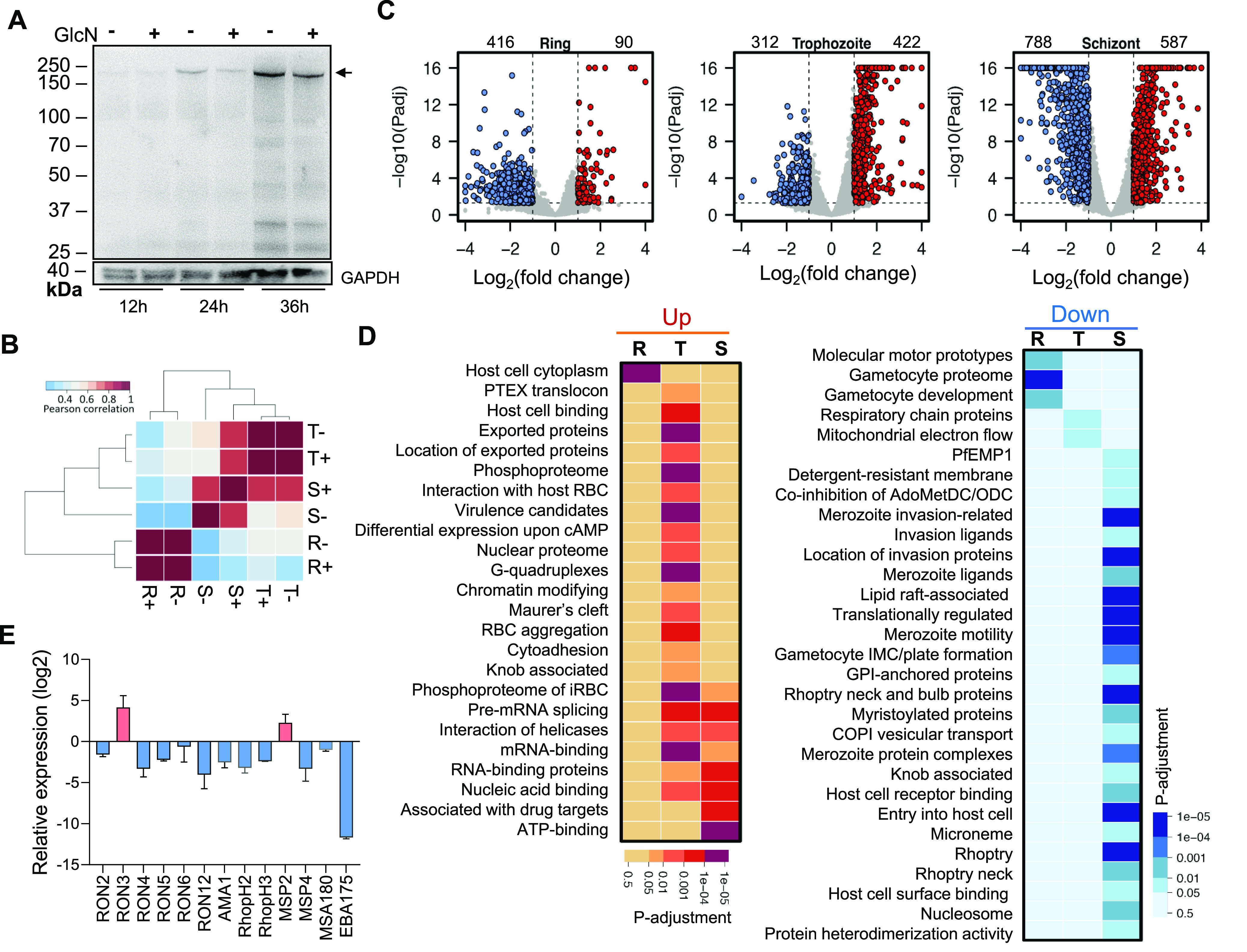
Global transcriptional analysis of GlcN-treated and -untreated NIF4^iKD^ parasites by RNA-seq. (A) Induced knockdown of PfNIF4-Ty protein in the NIF4^iKD^ parasites. Proteins were extracted from tightly synchronized NIF4^iKD^ parasites collected at 12, 24, and 36 hpi, respectively. Cultures were maintained with (+) or without (–) 2.5 mM GlcN supplement beginning at 3 hpi. Arrow indicates the recombinant NIF4-Ty protein band detected with the anti-Ty MAb. GAPDH was used as the control for equal protein loading. Representative images of three independent experiments are shown. (B) Pearson correlation coefficient of the transcriptomes for the ring (R), trophozoite (T), and schizont stages (S) of the NIF4^iKD^ parasite treated with (+) or without (–) 2.5 mM GlcN. (C) Volcano plots displaying the log_2_ (fold change) for GlcN+/GlcN– versus the –log_10_ *P* values at the ring, trophozoite, and schizont stages of the NIF4^iKD^ parasites. Each point represents a P. falciparum gene. The significantly differentially expressed genes (*p*-adj < 0.05 and >2-fold difference) are highlighted in red (upregulated genes) and blue (downregulated). The numbers of the differentially expressed genes are shown above the plots. (D) Gene ontology (GO) enrichment analysis of upregulated (left panel) and downregulated (right panel) genes in the NIF4^iKD^ parasites upon GlcN treatment at the ring (R), trophozoite (T), and schizont (S) stages, respectively. The color density indicates the level of significance (*p*-adj value) of the enriched GO term. (E) qRT-PCR validation of the expression of invasion-related genes in GlcN-treated parasites compared with untreated parasites. Tightly synchronized parasites were collected at the schizont stage (36 hpi). Error bars indicate SD from three biological replicates.

To understand the molecular basis of phenotypic changes from PfNIF4 KD, we analyzed the transcriptomes of GlcN-treated (GlcN+) and untreated (GlcN–) NIF4^iKD^ lines during the IDC. Tightly synchronized parasites at the ring (12 hpi), trophozoite (24 hpi), and schizont (36 hpi) stage were collected during the second IDC after the GlcN treatment for Western blot and RNA-sequencing (RNA-seq) analysis. Western blot analysis showed that GlcN treatment did not cause a noticeable change in the PfNIF4 level at the ring stage, while it only led to an 8.1% reduction of PfNIF4 at the trophozoite stage. However, GlcN treatment resulted in 35.2% KD of the PfNIF4 protein level at the schizont stage (Fig. 5A). We performed RNA-seq analysis at the corresponding time points of the IDC, each with three biological replicates. Hierarchical clustering analysis of the GlcN+ and GlcN– samples using the fragments per kilobase of transcript per million mapped reads (FPKM) values demonstrated stage-specific high correlations (Pearson correlation r^2^ = 0.886 to 1), indicating high reproducibility of the data (Fig. 5B). Compared with the untreated parasites, HTSeq analysis identified 90, 422, and 587 upregulated, and 416, 312, and 778 downregulated genes in GlcN-treated parasites at the ring, trophozoite, and schizont stages, respectively (Fig. 5C; Table S2A), indicating a more substantial effect of PfNIF4 KD at the schizont stage when PfNIF4 peak expression occurs. Gene ontology (GO) enrichment analysis showed that host cell cytoplasm and gametocyte development were enriched in the up- and downregulated categories at the ring stage, respectively (Fig. 5D; Table S2B). At the trophozoite stage, the majority of enriched GO categories were among the upregulated genes, including protein export (e.g., PTEX translocon, Maurer’s cleft, exported proteins), virulence (e.g., host cell binding, knob-associated proteins, cytoadhesion), and RNA metabolism (e.g., splicing, helicases, RNA binding, CCR4-NOT complex). In line with PfNIF4 being a protein phosphatase, PfNIF4 KD also led to the upregulation of the phosphoproteome in the trophozoites. Among the downregulated genes in trophozoites, only energy metabolism associated with the mitochondria was identified as the enriched GO category (Fig. 5D; Table S2B). It is also noteworthy that some of these genes, such as the mitochondrial ATP synthase F_0_ and F_1_ subunits and associated proteins, were significantly downregulated at the ring stage in PfNIF4 KD parasites (Table S2A). At the schizont stage, genes involved in RNA metabolism and proteins associated with drug targets were enriched among the upregulated genes, whereas 52% (13/25) of the highly enriched terms among the downregulated genes pertain to the invasion process (e.g., invasion ligands, merozoite motility, rhoptry, rhoptry neck, microneme), consistent with the reduced erythrocyte invasion upon PfNIF4 KD (Fig. 5D; Table S2B). To verify the effect of PfNIF4 KD on the expression of invasion-related genes, we selected 13 genes encoding proteins localized in the rhoptry, rhoptry neck, microneme, and merozoite surface for RT-PCR analysis. The results showed that 11 of the 13 genes analyzed displayed lower expression levels upon treatment with 2.5 mM GlcN at the schizont stage (Fig. 5E).

10.1128/mbio.01897-22.7 TABLE S2 Transcriptome analysis of NIF4^iKD^ parasites cultured with (GlcN+) or without GlcN (GlcN−) supplement. Download Table S2, XLSX file, 0.3 MB. Copyright © 2022 Zhu et al. 2022 Zhu et al. https://creativecommons.org/licenses/by/4.0/ This content is distributed under the terms of the Creative Commons Attribution 4.0 International license.

### PfNIF4 knockdown leads to downregulation of invasion- and motility-related protein expression.

**FIG 6:**
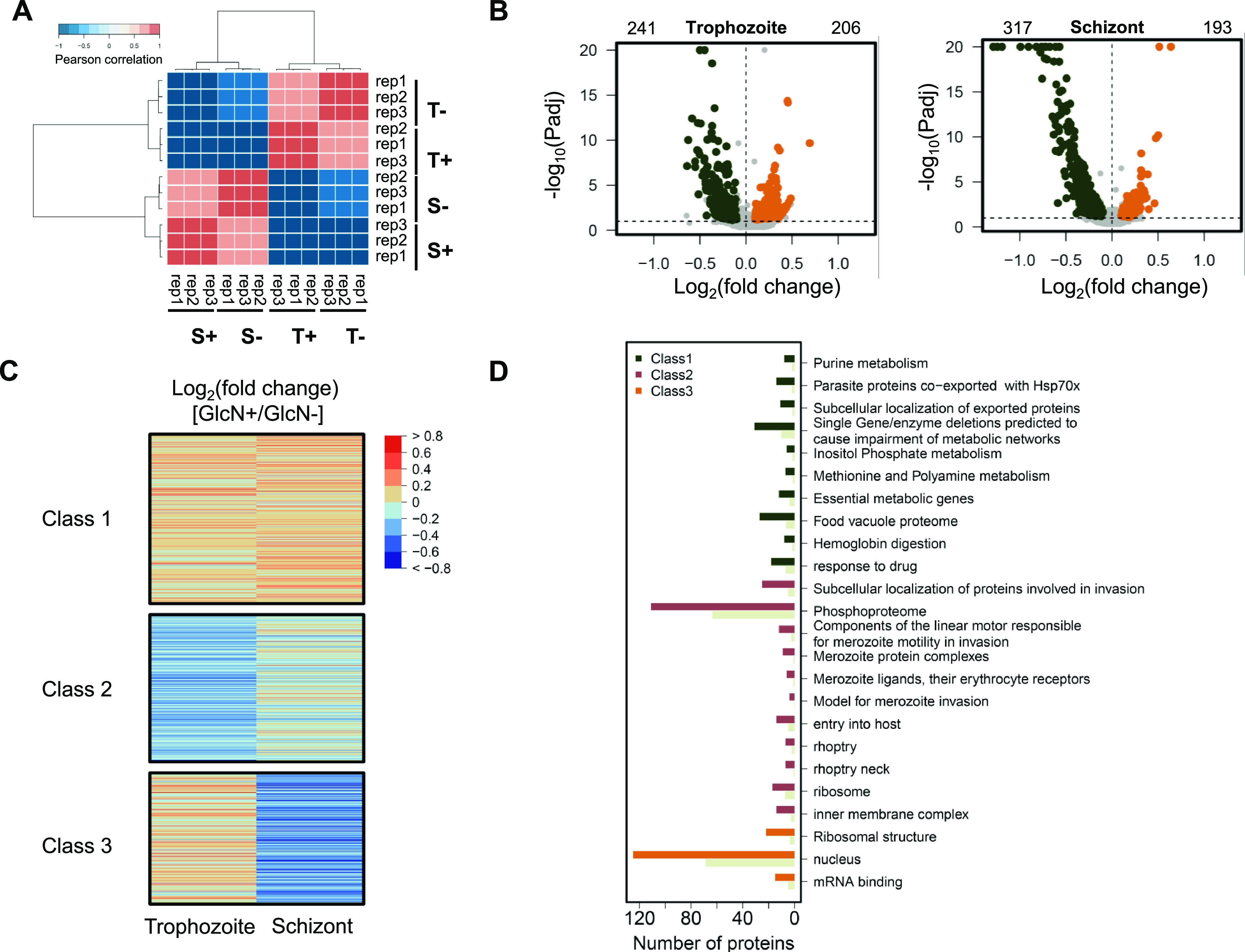
Global proteome analysis of GlcN-treated and -untreated NIF4^iKD^ parasites. (A) Heatmap displaying pairwise Pearson correlations of quantified proteomes of biological triplicates. (B) Volcano plot depicting the log_2_ (fold change) of PfNIF4^iKD^ parasites grown + or – GlcN at trophozoite and schizont stages in the second IDC cycle. Significantly up- and downregulated proteins are highlighted in orange and dark green, respectively. The numbers of the differentially expressed proteins are shown above the plots. (C) Heatmap displaying global changes in protein expression caused by PfNIF4 KD at the trophozoite and schizont stages. The proteins are organized according to the similarity in expression by K-means and combined into three classes. (D) Gene ontology (GO) enrichment analysis of dysregulated proteins in the NIF4^iKD^ parasites upon GlcN treatment. The three classes by K-means are indicated by different colors. The gray color indicates the expected number of proteins as the fraction of proteins in the whole genome.

To study the effect of PfNIF4 KD on protein expression, we performed proteomic analysis by liquid chromatography and tandem mass spectrometry (LC-MS/MS) using proteins collected at the trophozoite (24 ± 3 hpi) and schizont (36 ± 3 hpi) stages of synchronized NIF4^iKD^ parasites grown in the presence or absence of GlcN. High levels of correlation (Pearson *r* > 0.88 for each biological pair) were observed within each stage (Fig. 6A). Compared with the GlcN– samples, GlcN treatment led to 447 and 510 proteins being significantly dysregulated at the trophozoite and schizont stages (p-adjustment < 0.1 and log_2_ [fold change] uniformly higher or lower than 0 for all three biological replicates) (Fig. 6B; Table S3A). Proteins were predominantly downregulated in trophozoites (241) and especially in schizonts (317) (Fig. 6B; Table S3A). The dysregulated proteins were classified into three classes by *K*-means clustering (Fig. 6C). GO enrichment analysis revealed that genes in class 1 that were mainly upregulated at both trophozoite and schizont stages belong to metabolic process, response to drug, food vacuole proteome, hemoglobin digestion, and exported proteins GO terms (Fig. 6D; Table S3B). Proteins downregulated at the trophozoite stage (class 2) are enriched in GO terms associated with invasion and motility and phosphoproteome (Fig. 6D; Table S3B). In particular, proteins associated with the microneme (EBA175), rhoptry (RON2/4/5, RAP1/3, ROP14), merozoite surface (MSP1/2/7/9), glideosome (GAP40, GAP45, GAP50, GAPM1, GAPM2, ELC), and alveolates (IMC1c, IMC1f, IMC1g) showed different degrees of downregulation, with log_2_ (fold change) ranging from −0.51 to −0.01 at both trophozoite and schizont stages (Table S3A). Meanwhile, 22 large ribosomal subunits were significantly downregulated during schizogony (class 3) (Table S3A). These data strongly support the role of PfNIF4 in regulating the expression of invasion-related proteins and suggest that the invasion defect observed in PfNIF4 KD parasites is caused by the disturbed expression of invasion ligands.

10.1128/mbio.01897-22.8 TABLE S3 Proteome analysis of NIF4^iKD^ parasites grown + or − GlcN at trophozoite and schizont stages. Download Table S3, XLSX file, 0.4 MB. Copyright © 2022 Zhu et al. 2022 Zhu et al. https://creativecommons.org/licenses/by/4.0/ This content is distributed under the terms of the Creative Commons Attribution 4.0 International license.

### PfNIF4 interacts with RNAPII subunits and other chromatin factors.

To identify the putative NIF4 interaction candidates, we employed the proximity labeling technique TurboID, which exploits the activity of a mutant biotin ligase for protein biotinylation to allow robust purification of interacting proteins for proteomic analysis (28). We generated a transgenic parasite line, NIF4^TurboID^, by tagging the endogenous PfNIF4 protein with FKBP and TurboID using the selection-linked integration (SLI) system (29) (Fig. S3A). Correct integration of the plasmid into the *pfnif4* gene locus was verified by integration-specific PCR (Fig. S3B). Consistent with PfNIF4 being a nuclear protein, IFA using the anti-FKBP antibodies confirmed the predominant nuclear localization of PfNIF4-FKBP-TurboID fusion protein during the IDC (Fig. S3C). Further, after incubating the synchronized schizonts with 500 μM biotin for 10 min, TurboID-dependent protein biotinylation also detected nuclear localization of biotinylated proteins in the NIF4^TurboID^ parasite compared with the negative results in control 3D7 parasites (Fig. S3D).

10.1128/mbio.01897-22.3 FIG S3 Tagging of PfNIF4 with the TurboID and mass spectrometric analysis. Download FIG S3, PDF file, 0.3 MB. Copyright © 2022 Zhu et al. 2022 Zhu et al. https://creativecommons.org/licenses/by/4.0/ This content is distributed under the terms of the Creative Commons Attribution 4.0 International license.

To determine the putative PfNIF4-interacting proteins, biotinylated proteins were affinity-purified from the lysates of synchronized NIF4^TurboID^ schizonts using streptavidin magnetic beads and subjected to protein identification by LC-MS/MS. The 3D7 parasites were used as a negative control to detect background biotinylation (30, 31). The presence of the PfNIF4-FKBP-TurboID protein in the precipitated fraction was verified by Western blotting using the anti-FKBP antibody (Fig. S3E). The results of the proteomic analysis showed high reproducibility of the three biological replicates (Fig. S3F). A total of 2,812 unique peptides were identified and mapped to 985 proteins, 446 of which were quantifiable (Table S4A). Because PfNIF4 is a nuclear protein, the proteomic data were further compared with the P. falciparum nuclear proteome data set (32), which identified 200 nuclear proteins in the PfNIF4 interactome (Table S4B).

10.1128/mbio.01897-22.9 TABLE S4 Interactome analysis of PfNIF4 by TurboID. Download Table S4, XLSX file, 0.2 MB. Copyright © 2022 Zhu et al. 2022 Zhu et al. https://creativecommons.org/licenses/by/4.0/ This content is distributed under the terms of the Creative Commons Attribution 4.0 International license.

Using the label-free quantitation (LFQ) intensity algorithm, we identified a total of seven proteins with a cutoff of *P* value < 0.05 and log_2_ (fold change) > 1 as enriched in the NIF4^TurboID^ affinity purification compared to the 3D7 control (Table 2). Notably, these proteins include the RNAPII subunit (Rpb3), nucleosome components (histone H4, H3.3, and H3), and several 40S ribosomal proteins (Table 2). When protein abundance was calculated using intensity-based absolute quantification (iBAQ) and then normalized as an intensity-based fraction of total (iFOT), 24 proteins were identified in the affinity purification from NIF4^TurboID^ parasites but not from 3D7. These include ribosomal subunits, RNA splicing factors (RRP43 and SF3A1), chromatin-related (heterochromatin protein 1 [HP1] and GCN5), and an AP2-domain transcription factor (Table 2).

**TABLE 2:** Significantly enriched nuclear proteins identified in the NIF4^TurboID^ parasites by TurboID purification and mass spectrometry^*a*^

GeneID	Product description	Gene name or symbol	Log_2_fold change	Adjusted *P* value
PF3D7_0422400	40S ribosomal protein S19	RPS19	1.60	0.018
PF3D7_1105400	40S ribosomal protein S4, putative	RPS4	1.55	0.005
PF3D7_1105000	histone H4	H4	1.26	0.006
PF3D7_0617900	histone H3	H3.3	1.26	0.030
PF3D7_0610400	histone H3	H3	1.23	<0.001
PF3D7_0923000	DNA-directed RNA polymerase II subunit RPB3	RPB3	1.08	0.008
PF3D7_1302800	40S ribosomal protein S7, putative	N/A	1.03	0.011
PF3D7_0305600 ^*b*^	DNA-(apurinic or apyrimidinic site) endonuclease	APE1	1,000	<0.001
PF3D7_0413600	26S protease regulatory subunit 6B, putative	RPT3	1,000	<0.001
PF3D7_0520400	single-stranded DNA-binding protein, putative	N/A	1,000	<0.001
PF3D7_0527200	ubiquitin carboxyl-terminal hydrolase 14	USP14	1,000	<0.001
PF3D7_0604100	AP2 domain transcription factor	SIP2	1,000	<0.001
PF3D7_0703600	conserved protein, unknown function	N/A	1,000	<0.001
PF3D7_0706000	importin-7, putative	N/A	1,000	<0.001
PF3D7_0722400	Obg-like ATPase 1, putative	OLA1	1,000	<0.001
PF3D7_0802000	glutamate dehydrogenase, putative	GDH3	1,000	<0.001
PF3D7_0823300	histone acetyltransferase GCN5	GCN5	1,000	<0.001
PF3D7_0917600	pre-mRNA-splicing factor ATP-dependent RNA helicase PRP43, putative	PRP43	1,000	<0.001
PF3D7_1019400	60S ribosomal protein L30e, putative	N/A	1,000	<0.001
PF3D7_1103100	60S acidic ribosomal protein P1, putative	RPP1	1,000	<0.001
PF3D7_1220900	heterochromatin protein 1	HP1	1,000	<0.001
PF3D7_1244800	translation machinery-associated protein 46, putative	N/A	1,000	<0.001
PF3D7_1312900	eukaryotic translation initiation factor 4 gamma	EIF4G	1,000	<0.001
PF3D7_1317800	40S ribosomal protein S19	RPS19	1,000	<0.001
PF3D7_1319400	conserved protein, unknown function	N/A	1,000	<0.001
PF3D7_1408600	40S ribosomal protein S8e, putative	N/A	1,000	<0.001
PF3D7_1422800	actin-related protein ARP4	ARP4	1,000	<0.001
PF3D7_1426000	60S ribosomal protein L21	RPL21	1,000	<0.001
PF3D7_1434300	Hsp70/Hsp90 organizing protein	HOP	1,000	<0.001
PF3D7_1460700	60S ribosomal protein L27	RPL27	1,000	<0.001
PF3D7_1474500	splicing factor 3A subunit 1, putative	SF3A1	1,000	<0.001

a Proteins detected in both NIF4^TurboID^ IP and 3D7 control samples are calculated based on LFQ values, and candidates with a log_2_fold change value >1 are listed.

b Shaded are the proteins identified in the NIF4^TurboID^ IP but were absent from 3D7 control samples. All proteins and peptides have an FDR of <1%.

### Identification of PfNIF4 substrates by phosphoproteomics.

**FIG 7:**
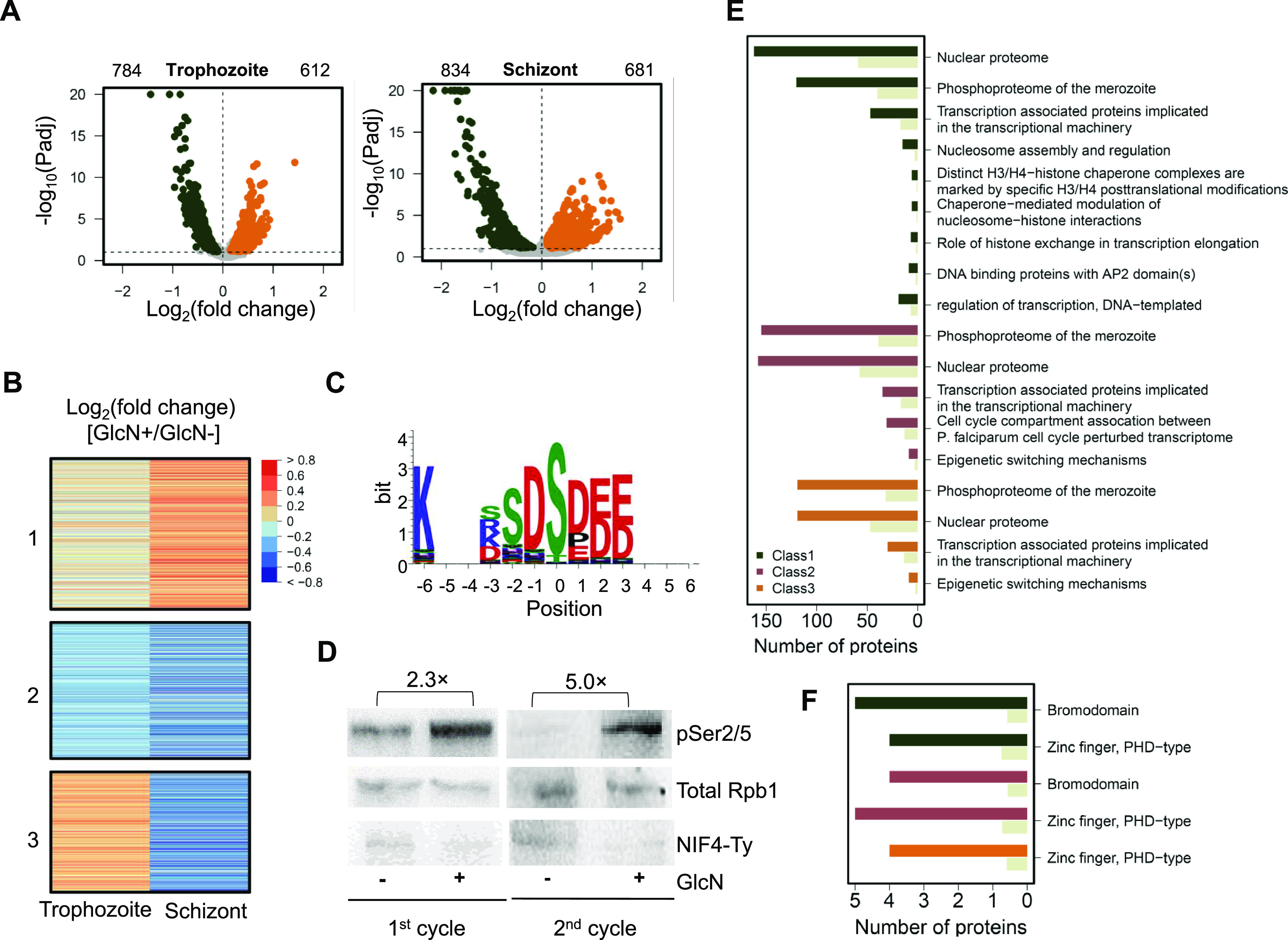
Phosphoproteome analysis of GlcN+ and GlcN– PfNIF4^iKD^ parasites. (A) Volcano plots showing proteins with differential phosphorylation levels in PfNIF4^iKD^ parasites (+) GlcN relative to (–) GlcN at trophozoite and schizont stages. Proteins with log_2_ (fold change) in phosphorylation levels higher or lower than 0 and adjusted *P* values of <0.1 are shown in orange (upregulated) and dark green (downregulated) dots, respectively. The numbers of respective proteins are indicated above the plots. (B) Heatmap displaying global changes of the protein phosphorylation levels caused by PfNIF4 KD at the trophozoite and schizont stages. The differentially regulated phosphoproteins are classified into three classes by K-means clustering. (C) Sequence logo showing consensus sequence surrounding phosphosites (position 0) significantly increased in the PfNIF4 KD parasites. (D) Immunoblots confirming the increased phosphorylation of Rpb1 CTD (pSer2/5) following PfNIF4 KD in parasites at 24 hpi in the first and second cycles. The band intensity was evaluated by Image J software. The fold increase (2.3× and 5.0×) indicates band intensity detected by the anti-Phospho-PfRpb1 CTD (Ser-2/5) antibody normalized to total Rpb1 detected with the anti-Rpb1 CTD (4H8) mouse MAb. The full Western blots are shown in Fig. S5. (E) GO enrichment analysis based on the three classes in (B). (F) Protein domain enrichment analysis of proteins classified into three classes. The gray bars in (E0 and (F) indicate the expected number of proteins as the fraction of proteins in the whole genome.

To identify potential substrates of PfNIF4 during asexual development, we carried out a quantitative phosphoproteomic analysis of GlcN+ and GlcN– NIF4^iKD^ parasites at the trophozoite and schizont stages. We induced KD at 3 hpi in tightly synchronized ring-stage parasites with a 3-h window and collected samples at the trophozoite (24 ± 3 hpi) and schizont (36 ± 3 hpi) stages in the second IDC. The proteome data showed high levels of correlation (Pearson *r* > 0.92 for each pair replicates) within each stage (Fig. S4A). We identified 6,354 phosphopeptides with 5,668 quantifiable phosphorylation sites that matched 1,224 quantifiable proteins (Fig. S4B; Table S5A), comparable with reported phosphoproteomic studies in P. falciparum (9, 25). Using criteria of *p*-adjustment < 0.1 and log_2_ (fold change) uniformly higher or lower than 0 for all three biological replicates, we identified 784 and 834 sites as downregulated, while 612 and 681 as upregulated in trophozoites and schizonts, respectively (Fig. 7A; Table S5B). Interestingly, the majority of the phosphopeptides with differential abundance belong to proteins known to be located in the nucleus (trophozoite, 64.9% [399/615]; schizont, 64.7% [408/631]), suggesting that PfNIF4 predominantly affects the protein phosphorylation of nuclear proteins.

10.1128/mbio.01897-22.4 FIG S4 Phosphoproteomic profiling of NIF4^iKD^ parasites. Download FIG S4, PDF file, 0.2 MB. Copyright © 2022 Zhu et al. 2022 Zhu et al. https://creativecommons.org/licenses/by/4.0/ This content is distributed under the terms of the Creative Commons Attribution 4.0 International license.

10.1128/mbio.01897-22.10 TABLE S5 (Related to FIG 7) Quantitative mass spectrometric global phosphoproteome analysis of GlcN+ and GlcN− NIF4^iKD^ parasitesTable S5, XLSX file, 2.7 MB. Copyright © 2022 Zhu et al. 2022 Zhu et al. https://creativecommons.org/licenses/by/4.0/ This content is distributed under the terms of the Creative Commons Attribution 4.0 International license.

10.1128/mbio.01897-22.5 FIG S5 Raw Western blot data related to FIG 7D. Download FIG S5, PDF file, 0.1 MB. Copyright © 2022 Zhu et al. 2022 Zhu et al. https://creativecommons.org/licenses/by/4.0/ This content is distributed under the terms of the Creative Commons Attribution 4.0 International license.

The differentially regulated phosphorylation sites were classified into three classes by K-means clustering (Fig. 7B; Table S5B). Peptides in class 1, primarily upregulated phosphorylation sites in trophozoites and schizonts, include 19 phosphorylation site motifs identified using the MOMO program from the MEME suite, which were significantly associated with PfNIF4 dephosphorylation (Table S5C). The consensus motif of PfNIF4 is shown in Fig. 7C. Of note, the serine-proline motif (xxxxxx_S_Pxxxxx), significantly enriched in class 1, resembles the mammalian consensus FCP1/SCP substrate sequence “YSPTSPS” and the “SP” motifs (33) found in CTD of other apicomplexans (Table S5C). Intriguingly, our phosphoproteome data revealed significantly upregulated phosphorylation levels on sites of “YSPTSPV” (S2352, 1.30-fold), “YSPYSIT” (S2367, 1.42-fold), and “FSPTSPA” (S2377, 1.30-fold) peptides in the heptad repeat-containing region (R2) of Rpb1 (Table S4).

To clarify whether PfNIF4 regulates RNAPII CTD phosphorylation, highly synchronized ring-stage NIF4^iKD^ parasites were treated with 2.5 mM GlcN at 3 hpi, and parasites were harvested at the late trophozoite stage (24 ± 6 hpi) in the first and second cycle, respectively. The phosphorylation level of the RNAPII CTD was evaluated by Western blotting analysis. The results showed that PfNIF4 KD led to a 2.3-fold (1st cycle) and 5.1-fold (2nd cycle) increase in phosphorylated Rbp1 at Ser2/5 of the heptad repeats compared to untreated parasites (Fig. 7D; Fig. S5).

GO analysis of class 1 proteins revealed significantly enriched molecular function GO terms, including nuclear proteome, phosphoproteome of the merozoite, and transcription-related (transcriptional machinery, AP2 proteins, and regulation of transcription) (Fig. 7E; Table S5C). Similarly, class 3 proteins with phosphorylation levels increased only in trophozoites were also enriched in transcription-related GO terms (e.g., transcription machinery and epigenetic factors) (Fig. 7E; Table S5D). Protein domain enrichment analysis of class 1 to 3 revealed that *bromodomain* (only observed in class 1) and PHD-type zinc-finger were significantly enriched, further supporting that PfNIF4 acts in dephosphorylation of transcriptional regulators in P. falciparum (*P* < 0.05) (Fig. 7F; Table S5E). In particular, class 1 and 3 proteins with significant changes in phosphorylation include many chromatin organization proteins (GCN5, H3, ISWI, SPT4, H2B, HP1, RUVB3, BDP2, PHD2, and ADA2), DNA-directed RNAP II subunit (RPB1, RPB2, RPB6, and RPB9), and AP2 domain transcription factors (ApiAp2, AP2-O5, SIP2, AP2-I), confirming that PfNIF4 affects the phosphorylation status of transcription regulators. Interestingly, proteins belonging to five epigenetic complexes, including the SIP2 complex (SIP2 [S943, 1.37-fold, trophozoite], PF3D7_1451200 [S1361, 1.20-fold, trophozoite and S605, 1.41-fold, schizont]), the BDP4 complex (CHD1 [S2842, 1.20-fold, trophozoite], PF3D7_1128000 [S1723, 1.51-fold, schizont], PF3D7_0306100 [S472, 1.11-fold, trophozoite]), the PHD2/SAGA-like complex (PHD2 [S1644, 1.36-fold, trophozoite and T4936, 1.17-fold, trophozoite], PF3D7_1019700 [S609, 1.22-fold, schizont], PF3D7_0817300 [S17, 1.30-fold, schizont and S1414, 1.37-fold, schizont]), and the TAF1/BDP5 complex (BDP5 [T2289, 1.35-fold, schizont, S2291, 1.35-fold, schizont, S2395, 1.52-fold, schizont, and T2397, 1.69-fold, schizont]) are enriched in significantly upregulated phosphosites in class 1 and/or 3 (Table S4B). These findings suggest that PfNIF4 may control the expression of virulence, drug resistance, and invasion-related genes by selectively dephosphorylating a number of transcriptional factors.

## DISCUSSION

In this study, we report the functional characterization of a nuclear protein phosphatase PfNIF4 of the NIF family in P. falciparum. PfNIF4 is essential for asexual propagation; its KD using the ribozyme KD system resulted in reduced merozoite invasion and increased susceptibility to ART drugs, but gametocytogenesis is unaffected. PfNIF4 KD led to substantial changes in transcriptomes and proteomes throughout the asexual IDC. Interactome analysis suggests that PfNIF4 may interact with chromatin factors and the RNAPII enzyme. Phosphoproteomic profiling identified a set of PfNIF4-related phosphoproteins with considerable functional similarity to Fcp/Scp substrates, particularly proteins involved in transcriptional regulation. Furthermore, Western blot analysis confirmed that PfNIF4 KD was associated with increased phosphorylation of the RNAPII CTD, suggesting an essential function of PfNIF4 in regulating transcription. These results are consistent with PfNIF4’s participation in the transcriptional regulation of several cellular pathways such as RNA metabolism, energy metabolism, stress responses, and RBC invasion.

The critical role of the NIF4 homolog FCP1 in facilitating transcription elongation and RNAPII recycling conforms to its requirement for viability in model eukaryotes such as the yeast and fruit fly (34, 35). Consistently, PfNIF4 is also essential for the asexual IDC, as demonstrated by its refraction to genetic deletion and *piggyBac* transposon insertion (36). Using the *glmS*-ribozyme system, we could achieve PfNIF4 protein KD by ~35% at the schizont stage, resulting in defects in asexual IDC, which is compatible with the constitutive expression of PfNIF4 during asexual development. Despite this modest PfNIF4 KD, global gene expression during the IDC was substantially disturbed, with significantly altered expression in 10% to 25% of the genes. Similarly, the inactivation of Fcp1p in yeast led to a global transcription shutdown (34). The interaction of Fcp/Scp family members with RNAPII is in line with their involvement in global gene regulation from yeast to mammalian cells (37, 38). Multiple lines of evidence also point to a similar mechanism by which PfNIF4 regulates global gene expression in malaria parasites. First, bioinformatic analysis identified that all *Plasmodium* NIF4s contain a conserved catalytic domain with high structural similarity to the yeast Fcp1p. Moreover, the catalytic domain bears a consensus motif DLDNT, where the two Asp residues are essential for dephosphorylating RNAPII CTD in yeast (34). Second, PfNIF4 was identified as a member of the RNAPII core complex in a large-scale protein-protein interaction network study and coclustered with several RNAPII subunits such as Rpb1, Rpb2, Rpb3, and Rpb9 (39). Using the TurboID approach, we also identified several RNAPII subunits in the PfNIF4 interactome, suggesting that they were located nearby in the parasite and may be functionally related. Finally, Fcp1 facilitates RNAPII elongation and recycling by modulating CTD phosphorylation (40). In yeast, Fcp1 specifically dephosphorylates Ser2/5 (41, 42). Similarly, we observed that PfNIF4 KD in P. falciparum was associated with increased phosphorylation of the second serine in the heptad repeat “YSPTSPV,” “YSPYSIT,” and “FSPTSPA” in Rpb1 CTD at the trophozoite stage by phosphoproteome analysis. Meanwhile, we also observed upregulated phosphorylation of Rpb1 CTD repeat region by immunoblotting with anti-Phospho-PfRpb1 (Ser2/5) antibody in trophozoites, suggesting a preference motif for PfNIF4 in Rpb1 CTD phosphorylation may be ‘(Y/F)SPTS(P/I)(K/V)’ in P. falciparum.

In line with the potential role in regulating RNAPII-mediated transcription, PfNIF4 peak expression coincides with the peak RNAPII-dependent transcriptional activity at the schizont stage during the IDC (43). It thus seems reasonable that the effect of PfNIF4 KD on global gene expression was most profound at the schizont stage (Fig. 5). Phenotypically, the modest extent of PfNIF4 KD did not affect the progression of the IDC, merozoite development, or egress, but only resulted in reduced merozoite invasion of RBCs. Erythrocyte invasion events are facilitated by multiple receptor-ligand interactions, including events such as protease cleavage, signal transduction, organelle release, and actin-myosin motor engagement (44). Consistent with this phenotype, downregulated genes in the schizonts are significantly enriched in those associated with RBC invasion, among which are those functionally characterized to play prominent roles at the different steps of invasion—merozoite surface proteins, microneme proteins (e.g., EBA175, AMA1), and rhoptry proteins (e.g., Rhoph2 and RhopH3) (45–50). Profiling RNAPII occupancy during the IDC identified a class of genes with peak RNAPII occupancy during late IDC stages, which are also enriched in invasion pathways (16). While specific recruitment of the RNAPII to invasion-related genes is mediated or influenced by transcriptional factors such as AP2-I (51), AP2-LT and its associated euchromatin modifier PfGCN5 (52), and the acetyl mark reader PfBDP1 (53), it is likely that PfNIF4 is concurrently recruited to these genes. In support of this assumption, our proximity-based interactome analysis identified copurification of PfNIF4 with GCN5 and AP2-I, although the latter did not achieve significance in enrichment. Interestingly, we also observed upregulated phosphorylation at the T854 site (1.2-fold) of PfGCN5 in PfNIF4 KD schizonts. The altered phosphorylation status of GCN5 may affect its interaction with the RNAPII complex or directly influence its function in activating gene transcription during schizogony. Meanwhile, the dysregulated phosphorylated CTD caused by PfNIF4 KD early in IDC may also abrogate RNAPII recycling in the transcription of invasion-related genes. Furthermore, our phosphoproteomic analysis also revealed a set of PfNIF4-regulated phosphoproteins, including ribosomal proteins and translation initiation factors in the nucleus, suggesting additional targets of PfNIF4 during schizogony (32, 54). Notably, Fcp1 has been found to dephosphorylate other targets in mammals and yeast (55–58), suggesting a more profound role of this family of proteins beyond regulating RNAPII-CTD. Further studies are necessary to comprehensively identify the PfNIF4 substrates and dissect the molecular mechanisms by which NIF4-dependent dephosphorylation regulates transcription in *Plasmodium*.

While the invasion-specific effect of PfNIF4 KD is consistent with the concurrent peak expression of PfNIF4 and invasion-related genes, we cannot exclude that PfNIF4 may be essential during the early IDC because only a low-to-modest level of PfNIF4 KD could be obtained at the ring and trophozoite stages. Nevertheless, genes upregulated at these early stages upon PfNIF4 KD are enriched in protein export and host cell interactions, many of which are located in heterochromatin regions and regulated epigenetically (59). We identified hyperphosphorylated CTD caused by PfNIF4 KD in trophozoites by both phosphoproteome and immunoblotting. Aside from the CTD phosphatase function of PfNIF4, we also observed significantly upregulated phosphosites in epigenetic regulators, such as PfSIP2 (S943) at the trophozoite stage upon PfNIF4 KD. Furthermore, the identification in the PfNIF4 interactome of PfSIP2, which binds to cis-regulatory elements in *var* promoters to promote heterochromatin formation and *var* gene silencing (60), and the heterochromatin marker HP1 (61), supports the involvement of PfNIF4 in regulating the variant gene families. RNAPII CTD dephosphorylation by PfNIF4 may establish a platform for PfSET2 binding (62), which deposits the H3K36me2 mark at telomeres and multicopy variant gene families (63). It is noteworthy that *PfSET2* was also upregulated by >2.5-folds in both trophozoites and schizonts upon PfNIF4 KD. In addition, major epigenetic transcriptional factors, including PF3D7_1451200, BDP4 complex members, PHD2/SAGA-like complex, and TAF1/BDP5, are also hyperphosphorylated in PfNIF4 KD parasites. Although we did not find evidence for direct interactions between PfNIF4 and these transcriptional factors, previous studies reported that these epigenetic factors interact with GCN5/ADA2 complex during transcriptional initiation (53, 64–66). Therefore, it is tempting to speculate that these transcriptional cofactors, such as GCN5, may direct PfNIF4 to dephosphorylate these substrates, which may directly or indirectly (through RNAPII complex) affect their functions in transcriptional initiation. Among the upregulated genes during the IDC, GO categories involved in RNA metabolism (e.g., splicing, RNA-binding) are also enriched, suggesting the possible influence of PfNIF4 KD on mRNA stability and translation. The phosphoproteomic GO enrichment analysis also reflects an enrichment of the “inner membrane pellicle complex” GO term in upregulated phosphoproteins in trophozoites, including the glideosome components (GAP45, GAP40) and alveolins (IMC1c, IMC1f, and IMC1g). One possible explanation for this observation is that PfNIF4 KD may selectively activate a protein kinase to phosphorylate these proteins. A previous phosphoproteomic analysis of cdc2-related protein kinase 4 (CRK4) revealed putative substrates, including GAP45, GAP40, IMC1g and IMC1c, and shared 29% (5/17) identical regulated phosphosites with our PfNIF4-regulated phosphoproteome (67). Interestingly, we observed significantly increased phosphorylation at S1032 and S441 sites of CRK4 protein upon PfNIF4 KD (Table S5). Phosphorylation of CRK4 at S1032 and S441 sites may be essential for its full kinase activity for substrates such as glideosome and alveolins, although more experiments are needed to test this hypothesis.

Finally, our data support the potential involvement of PfNIF4 in ART resistance. GWAS has identified a mutation (V1157L) in PfNIF4 that was strongly associated with clinical ART resistance in the eastern GMS (21). The field parasite population from the China-Myanmar border also harbors the Y1133N and V1157L mutations, which were associated with reduced *in vitro* susceptibility to DHA and artemether (22). Interestingly, PfNIF4 KD led to increased susceptibility to ARTs, as shown in reduced RSA values and lower IC_50_ values to DHA and artemether. This increased ART sensitivity phenotype might be associated with transcriptomic changes in mitochondrial activity, lipid metabolism, and hemoglobin uptake pathways. Mitochondrial activity is critical for the survival of parasites after ART exposure (68). The mitochondrial electron transfer chain components in ART-resistant parasites are upregulated, enabling higher ATP biosynthesis upon exposure to ARTs (69). In the “persister” rings after ART exposure, mitochondria are enlarged, closely juxtaposed with the nucleus, and associated with an altered metabolic state (70). In the PfNIF4 KD parasites, the GO categories “respiratory chain proteins” and “mitochondrial electron flow” were significantly enriched in trophozoites, while the mitochondrial membrane ATP synthase subunits (F0, F1, and several associated proteins) were also downregulated by more than two folds at the ring stage (Table S2A). These transcriptomic changes may translate into a decreased metabolic state of the mitochondria, partially contributing to the increased ART sensitivity. In defining the K13-mediated ART resistance mechanism, K13 was found to associate with a dozen of proteins, including ubiquitin carboxyl-terminal hydrolase 1 (UBP1), metacaspase-2, Eps15-like protein, and other K13-interacting candidates (KICs), and the KD of eight of these proteins rendered parasites resistant to ART (71). Intriguingly, in the PfNIF4 KD parasites, UBP1, Eps15, KIC1, 2, 4, 5, 6, and 9 were upregulated in trophozoites, whereas several of them were downregulated in schizonts (Table S2A). Meanwhile, we also observed enrichment of GO terms, such as “food vacuole proteome” and “hemoglobin digestion” in class 1 of our proteome analysis, suggesting the digestive vacuole biogenesis and the uptake/degradation of hemoglobin are affected in PfNIF4 KD parasites. Altered expression of these genes in PfNIF4 KD parasites may interfere with endocytosis, resulting in changed sensitivity to ART (72). Another change resulting from PfNIF4 KD is the downregulation of the GO terms “lipid-raft-associated” and “detergent-resistant membrane,” which may also be associated with altered ART sensitivity. K13-mediated ART resistance has been found to correlate with the elevation of phosphatidylinositol-3-phosphate (PI3P) and dramatically increased PI3P vesicles (73, 74). In light of these findings, it will be interesting to determine if the PfNIF4 mutations would alter protein-protein interaction and PfNIF4 activity, thus affecting the transcription of factors involved in ART resistance.

## MATERIALS AND METHODS

### Sequence analysis.

The *pfnif4* (PF3D7_1012700) genomic sequence and orthologs of other *Plasmodium* species were retrieved from PlasmoDB (http://www.plasmodb.org). The CPDc domain and BRCT domain were analyzed by SMART (http://smart.embl-heidelberg.de/). The schematic figure of NIF4 proteins was drawn using the IBS 1.0.3 software (75). The three-dimensional (3D) structures of PfNIF4 and its putative catalytic domain were predicted by using the I-TASSER program (76). Multiple sequence alignment of the CPDc domain in NIF4 proteins was performed using the RPS-BLAST program of NCBI conserved domains search (https://www.ncbi.nlm.nih.gov/Structure/cdd/wrpsb.cgi). Sequence identity was calculated by using the Clustal Omega program (http://www.uniprot.org/align/). The phylogenetic analysis of the NIF4 orthologs was performed using MEGA X and Eolview v3 software (77, 78).

### Plasmid construction.

To generate the pL6-cs-NIF4^iKD^ plasmid for CRISPR/Cas9, the *pfnif4* fragment (from 3,973 to +500 bp) used for homologous recombination was amplified from the 3D7 genomic DNA using primer pairs NIF4^iKD^-AsciF1/NIF4^iKD^-R1 and NIF4^iKD^-F/NIF4^iKD^-AflII1 (Table S1). The primers bear the NheI/StuI sites (underlined) and 15-bp overlapping sequences necessary for InFusion cloning using the restriction sites *Asc*I/*Afl*II. Then, the triple Ty and *glmS* sequences (3×Ty-*glmS*) were amplified using primers 3Ty+*glmS*.F and 3Ty+*glmS*.R and cloned into the NheI and StuI sites of the bypass plasmid, pL6-cs-NIF4-HR (Table S1). PCR amplifications were done with KOD-plus neo (Toyobo, Osaka, Japan) and cloned into the Escherichia coli XL10-Gold Ultracompetent Cells (Clontech) using the In-Fusion HD Cloning Kit (Clontech, CA, USA). Guide-RNA cloning also used the In-Fusion HD Cloning Kit (Clontech), with the 20-bp guide RNA surrounded by the 15-bp overlapping sequence from the pl6-cs plasmid necessary for InFusion cloning (Table S1). The pUF1-BSD-Cas9 plasmid with the BSD selection cassette modified based on pUF1-Cas9 was used for Cas9 expression (79). To tag the *pfnif4* gene with the FKBP-TurboID tag (28), the GFP gene in the pSLI-2×FKBP-GFP plasmid (a gift from Tobias Spielmann) was replaced with the TurboID coding sequence with the primers listed in Table S1 using an In-Fusion HD Cloning Kit (Clontech) to generate the pSLI- 2×FKBP-TurboID plasmid. Then the C-terminus (3,707 to 4,574 bp) of the *pfnif4* gene was amplified with primer pairs NIF4-Cter.F and NIF4-Cter.R (Table S1) and ligated into the NotI and AvrII sites of the modified plasmid to generate the pSLI-PfNIF4-2×FKBP-TurboID plasmid. For parasite transfection, all constructs and control plasmids were purified using a Maxiprep kit (Qiagen, Dusseldorf, Germany).

10.1128/mbio.01897-22.6 TABLE S1 Primers used in this study. Download Table S1, DOCX file, 0.02 MB. Copyright © 2022 Zhu et al. 2022 Zhu et al. https://creativecommons.org/licenses/by/4.0/ This content is distributed under the terms of the Creative Commons Attribution 4.0 International license.

### Parasite culture and transfection.

P. falciparum-infected human erythrocytes were maintained in the RPMI 1640 culture medium containing O^+^ human RBCs at 2.5% hematocrit supplemented with 0.5% Albumax I (Thermo Fisher, Waltham, USA), 0.2 mM hypoxanthine (Sigma-Aldrich, MO, USA), and 10 μg/L gentamicin (Sigma-Aldrich) under 5% O_2_/5% CO_2_/90% N_2_ at 37°C according to standard procedures (80). Synchronized parasites were obtained by 5% sorbitol treatment of ring-stage culture and/or 40%/70% Percoll enrichment of schizonts (81). Transfection of the 3D7 parasite was done by electroporation of ~50 μg of pUF1-BSD-Cas9 and 50 μg plasmid DNA of pL7-cs-NIF4^iKD^ into uninfected RBCs in a 2-mm cuvette using the Gene Pulser Xcell Electroporation System (Bio-Rad, CA, USA). Parasites were selected with 2.5 nM WR99210 (Jacobus Pharmaceuticals) and 2 μg/mL Blasticidin S (Thermo Fisher) for ~3 weeks with weekly replenishment of fresh RBCs until resistant parasites were seen. Single clones of parasites with stable integration of the constructs were obtained by limiting dilution (82). Correct editing of the *pfnif4* gene locus in the genetically manipulated parasites was verified by PCR and Western blotting (Table S1). Four clones from two different transfection experiments were randomly selected to characterize the integration events, and two were used for phenotypic analysis.

### Western blotting.

The parasite samples were released from erythrocytes with 0.15% saponin (Sigma-Aldrich) in cold phosphate-buffered saline (PBS, pH 7.4). Parasite proteins were extracted with the M-PER Mammalian Protein Extraction Reagent (Thermo Fisher) for 30 min on ice. For phosphatase treatment, a final concentration of 2.5U mL^−1^ alkaline phosphatase (Sigma-Aldrich) was added to the NIF4^iKD^ parasite lysates and incubated for 1 h with the addition of 1 mM MgCl_2_ at 37°C. Protein concentrations were measured using the Pierce BCA Protein assay kit (Thermo Fisher). Parasite extracts were boiled under reducing conditions in the NuPAGE LDS sample buffer (4×) (Thermo Fisher), and equal amounts of proteins (50 μg) were separated by 10% SDS-PAGE. Separated proteins were transferred to a 0.22 μm PVDF membrane (Millipore, Bedford, USA) and probed with the anti-Ty1 MAb (Thermo Fisher), or anti-Phospho-Rpb1 (Ser-2/5) (Abcam), or anti-Rpb1 CTD (Abcam) at 1:1,000 in Tris-buffered saline containing 0.1% Tween 20 (TBS-T) for 2 h at room temperature, followed by incubation with the horseradish peroxidase (HRP)-conjugated antibodies at 1:20,000 (Thermo Fisher) in TBS-T for 1 h. As a loading control, the membrane was probed with antibodies against the heat shock protein 70 (Hsp70, Abcam) or GAPDH (Abcam) at 1:500 in TBS-T. The detected proteins were visualized using an enhanced chemiluminescence kit (Invitrogen, MA, USA) on Tanon 4200 (Tanon, Shanghai, China). The relative levels of the Ty-tagged PfNIF4 were estimated using a densitometer by Image J software.

### Fluorescence microscopy.

The subcellular localization of NIF4-Ty in P. falciparum was observed under a fluorescence microscope as previously described (83). Briefly, the NIF4^iKD^ parasites were fixed with 4% paraformaldehyde/0.0075% glutaraldehyde in PBS at room temperature for 30 min. Fixed parasites were washed with PBS three times, permeabilized with 0.1% Triton X-100 in PBS for 10 min on ice, rinsed with 0.1 mg/mL of sodium borohydride in PBS, and blocked in 5% skimmed milk. Then, the samples were incubated with mouse anti-Ty1 MAb (1:500), the rabbit anti-EXP2 sera (1: 500) as the PVM marker, or the rabbit anti-Pfs16 sera (1:500) as the gametocyte marker. Secondary antibodies (Alexa Fluor 488 goat anti-mouse IgG or 594 anti-rabbit IgG) (Thermo Fisher) were used at 1:500 dilution. The samples were mounted with ProLong Diamond Antifade Mountant with 4’,6-diamidino-2-phenylindole (DAPI, Thermo Fisher) and observed by Nikon C2 fluorescence confocal laser scanning microscope. Postprocessing and image analysis were performed using Adobe Photoshop and Image J software. At least 20 images were captured for each colocalization experiment, and Pearson’s correlation coefficients were calculated.

### Growth phenotype analysis.

All phenotype assays were performed in three replicates, and the slide reading was done blinded. To compare parasite’s progression through the IDC, schizont-stage parasites from tightly synchronized cultures were purified on a 45%/75% Percoll gradient and allowed to rupture and invade erythrocytes for 3 h. Unruptured schizonts were eliminated using 5% sorbitol treatment. Synchronized ring-stage parasites (1 mL with or without 2.5 mM GlcN treatment) were seeded at 1% parasitemia and 2% hematocrit in a 24-well plate, and progression of parasites through the IDC was monitored by Giemsa-stained thin smears every 2 h. To measure parasite proliferation, the tightly synchronized ring-stage cultures (1 mL with or without 2.5 mM GlcN treatment) were initiated at 0.1%, and the medium was changed daily for a week. Giemsa-stained thin smears were made to determine the daily parasitemia by counting parasites in at least 3,000 RBCs.

For the invasion and egress assays, tightly synchronized 3D7 or NIF4^iKD^ schizonts were incubated with RBCs at 1% parasitemia and 2.5% hematocrit. After 12 h of incubation, the numbers of rings and schizonts were determined by counting at least 1,000 cells per condition in randomly selected fields on Giemsa-stained thin smears. The numbers of ruptured schizonts and new rings formed per ruptured schizont were calculated with the following equations: ruptured schizonts = ([no. of schizonts at 0 h/no. of RBCs at 0 h] – [no. of schizonts at 12 h/no. of RBCs at 12 h])/[no. of schizonts at 0 h/no. of RBCs at 0 h]; Number of rings per ruptured schizont = ([no. of rings at 12 h/no. of RBCs at 12 h] – [no. of rings at 0 h/no. of RBCs at 0 h])/([no. of schizonts at 0 h/no. of RBCs at 0 h] – [no. of schizonts at 12 h/no. of RBCs at 12 h]). In addition, the RBC invasion assay was also performed using merozoites purified mechanically through filtration from E64-treated schizonts after 52 h (84).

To determine the role of NIF4 in gametocytogenesis, gametocyte induction was performed as described previously with slight modifications. Briefly, synchronous schizonts at a parasitemia of 0.3% were incubated in 15 mL of complete medium with 6% hematocrit at 37°C. This setting-up day was regarded as the first day of postgametocyte induction. Subsequently, the medium was changed daily and increased to 25 mL when the parasitemia reached 5%. The culture was maintained for 12 days until gametocytes became mature. The asexual parasites were eliminated by treatment with 10 U/mL heparin (Dingguo, Beijing, China) from day 5 postgametocyte induction when the induced parasites were at the ring stage and the parasitemia reached about 10% to 13%. Induced KD of NIF4 protein was performed by supplementing the culture with 2.5 mM GlcN from day 5 to 12 postgametocyte induction. Gametocytes were determined by staining with the anti-Pfs16 antibodies (Genescipt Co. Nanjing, China), and gametocytemia was counted per 5,000 RBCs. Gametocyte stages at different time points were recorded. Mature male and female gametocytes were differentiated using morphological characteristics of mature gametocytes in Giemsa-stained thin films (85). The sex ratio of male to female was determined by counting at least 500 gametocytes on each slide.

### *In vitro* drug assays.

The *in vitro* susceptibilities of NIF4^iKD^ parasites (with or without 2.5 mM GlcN treatment) to 10 antimalarial drugs, amodiaquine dihydrochloride dihydrate, artemether, artesunate, DHA, chloroquine, piperaquine, mefloquine, quinine, lumefantrine, and pyronaridine were assayed using the SYBR green I-based assay (22). Artemether, artesunate, DHA, and piperaquine were purchased from Kunming Pharmaceutical Co. (Kunming, Yunnan, China), while the rest of the drugs were purchased from Sigma-Aldrich (St. Louis, MO, USA). The stock solutions were prepared as described before (22). The 5% sorbitol synchronized ring-stage 3D7 and NIF4^iKD^ parasites were diluted with the complete medium and O^+^ RBC to 1% hematocrit and 0.5% parasitemia and cultured with the serially diluted concentrations of each drug in a 96-well microplate. Wells with no drug and only RBCs were used as a positive control and the background, respectively. For each antimalarial drug, two technical repeats and three biological repeats were performed. The fluorescence intensities of the plate were measured using a TECAN Spark 10M plate reader (BMG Labtech Inc, Ortenberg, Germany) at excitation and emission wavelengths of 485 and 520 nm, respectively. The IC_50_ value of each antimalarial drug was calculated using the GraphPad Prism 8.0.1 program.

RSA_0–3h_ was conducted as previously described (86, 87). Briefly, highly synchronous 3D7 and NIF4^iKD^ parasites cultures at the early ring stage (0 to 3 hpi) at 1% parasitemia and 2% hematocrit were exposed to either 700 nM DHA or DMSO for 6 h, followed by three times washing with RPMI 1640 medium, and cultured with complete medium for 66 h. For *in vitro* sensitivity assays of the 3D7 and NIF4^iKD^ parasites to a gradient of DHA concentration, tightly synchronized early rings (0 to 3 hpi) were exposed to serial dilutions of DHA (1.4 to 700 nM), which were prealiquoted into 96-well plates, at 0.5% parasitemia, 2% hematocrit, and 200 μL final volume per well. Triplicate wells were included for each parasite line and drug concentration. The vehicle control for the RSA was DMSO. Following a 3-h drug incubation, parasites were washed four times with RPMI 1640 medium and cultured in the fresh medium until the assay endpoint of 72 h. For both the RSA_0 – 3h_ assays (700 nM DHA and 1.4 to 700 nM DHA), parasitemia was assessed by estimating 10,000 RBCs from Giemsa-stained blood films. Percentage parasite survival was calculated as the parasitemia in the presence of drug divided by the parasitemia in the vehicle control well, multiplying the result by 100.

### RNA extraction, RNA-seq, and RT-PCR.

Samples for RNA-seq analyses were taken at 12, 24, and 36 hpi from highly synchronized cultures. For RNA analyses, erythrocyte pellets were rapidly lysed in 10× volumes of prewarmed TRIzol (Thermo Fisher, Waltham, USA) and stored at −80°C until RNA purification. The experiment was repeated at 4-week intervals to obtain three RNA samples as biological replicates. RNA purification and DNase treatment of the samples were performed as described previously (88). mRNA was purified from total RNA using poly-T oligo-attached magnetic beads. The clustering of the index-coded samples was performed on a cBot Cluster Generation System using TruSeq PE Cluster Kit v3-cBot-HS (Illumia) according to the manufacturer’s instructions. After cluster generation, the library preparations were sequenced on an Illumina platform with paired-end 150 bp read chemistry. Raw reads in fastq format were first processed through in-house Perl scripts. The UMI (Unique Molecular Identifiers) was extracted by UMI-tools v1.0.0 (89). All the downstream analyses were based on high-quality UMI reads (89). Strand-specific RNA-seq paired-end reads were mapped onto the P. falciparum 3D7 genome (assembly GCA_000002765) using Hisat2 v2.0.4 (90). UMI-tools v1.0.0 were used to deduplicate reads based on the mapping coordinates and the UMI attached to the reads (89). HTSeq v0.9.1 was used to count the reads mapped to each gene. The FPKM of each gene was calculated based on the length of the gene and reads count mapped to this gene. Differential expression analysis of genes between the WT and NIF4^iKD^ lines (three biological replicates per condition) was performed using the DESeq R package (1.18.0). The resulting *P* values were adjusted using Benjamini and Hochberg’s approach for controlling the false discovery rate. Genes with an adjusted *P* value <0.05 found by DESeq were assigned as differentially expressed. GO enrichment analysis of differentially expressed genes was implemented using the GOseq R package, in which gene length bias was corrected (91). GO terms with corrected *P* value less than 0.05 were considered significantly enriched by differentially expressed genes. The RNA-seq raw data were deposited in the NCBI via the GEO (accession number: GSE208865).

RT-PCR was performed on tightly synchronized schizont-stage (36 hpi) parasites for several invasion-related genes (primers listed in Table S1). Isolated RNA was treated with DNase I (Promega) and used in reverse transcription reactions (SuperScript III Reverse Transcription kit, Invitrogen) with 500 ng of total RNA. cDNA was diluted 1:20 with DEPC-treated water before use. Gene expression was quantified by SYBR green PCR using TB Green Premix *Ex Taq* II (TaKaRa) on an CFX96^TM^ Real-Time System (BIO-RAD). Reaction mixtures were incubated at 95°C for 15 min and then subjected to 40 cycles of 95°C for 15 s and 60°C for 1 min with a subsequent melting step (60 to 95°C). The constitutively expressed *seryl-tRNA synthetase* (PF3D7_0717700) gene was used as an internal reference using primers PF3D7_0717700F and PF3D7_0717700R (Table S1). All expression values were further normalized with the respective values in the transgenic parasites. Three biological replicates, each with three technical replicates, were performed for each assayed gene. The relative expression value of each gene was calculated as described previously using the 2^-ΔΔCt^ method (92, 93).

### Proteomic analysis.

Tightly synchronized NIF4^iKD^ parasite cultures (3-h window) with (+) or without (–) GlcN supplement at 3 hpi were harvested at the trophozoite (24 ± 3 hpi) and schizont (36 ± 3 hpi) stages in the second IDC by centrifugation at 400 × *g* for 10 min. The erythrocytes were lysed with 0.15% saponin (Sigma-Aldrich) for 10 min on ice. The parasites were pelleted and washed in ice-cold PBS containing a protease inhibitor cocktail (Thermo Fisher) and centrifuged at 1,700 × *g* for 10 min at 4°C to remove hemoglobin. The samples were sonicated three times on ice using a high-intensity sonicator (Scientz) in lysis buffer (8 M urea containing 1% protease inhibitor cocktail), and centrifuged at 14,000 × *g* for 10 min at 4°C to remove debris. The protein solution was reduced with 5 mM DTT (Sigma-Aldrich), alkylated with 11 mM iodoacetamide and digested with trypsin overnight at 37°C.

For isobaric labeling with tandem mass tags (TMT), peptides from each sample were dissolved in 0.5 M TEAB. Each peptide channel was labeled with its respective TMT reagent (Thermo Fisher) and incubated for 2 h at room temperature as previously described (94). After checking the labeling efficiency, the TMT-labeled samples were desalted with Strata X C18 SPE column (Phenomenex, Beijing, China) and dried by vacuum centrifugation. Protein preparation was performed with three biological replicates.

LC-MS/MS was performed as previously described (95). Briefly, the tryptic peptides were dissolved in solvent A (0.1% formic acid) and directly loaded onto a homemade reversed-phase analytical column (15 cm in length, 75 μm in diameter). Subsequently, peptides were eluted via the analytical column with a constant flow of 500 nL/min on an EASY-nLC 1200 Ultra Performance Liquid Chromatography (UPLC) system and introduced into the Q Exactive HF-X mass spectrometer (Thermo Fisher). A full mass scan was acquired with a mass range of 400 to 1500 *m/z* in profile mode with a resolution of 120,000. Peptides were then selected for MS/MS using the NCE setting at 28, and the fragments were detected in the Orbitrap at a resolution of 15,000. The resulting MS/MS data were processed using the MaxQuant version 1.6.15.0 (96). Tandem mass spectra were searched against a Uniprot P. falciparum (UP000001450) proteome database concatenated with a reverse decoy database. Trypsin/P was specified as the cleavage enzyme with an allowance of up to two missed cleavages. For the analysis of proteomic data, the precursor ion mass tolerance was set as 20 ppm in the first search and 4.5 ppm in the main search, and the mass tolerance for fragment ions was set as 0.02 Da. Carbamidomethylation of cysteines was specified as a fixed modification, and acetylation modification and oxidation of methionine were set as variable modifications. The false discovery rate (FDR) on peptide and protein levels was adjusted to 1%, and the minimum score for modified peptides was set to >40. Proteins were identified with a minimum of one unique peptide. The MS/MS raw data and peptide information, including peptide sequence, number of unique peptides and spectral count, percentage sequence coverate and Mascot score were deposited in the ProteomeXchange via the PRIDE database (PXD034167) for open access (97). The improved Gaussian model was implemented to capture global protein alteration after GlcN treatment. Similar methods have been successfully used in multiple studies (98–100) and recently applied to other Apicompexa parasites (101). Basically, the log_2_ (fold change) of normalized protein/peptide intensities were modeled by the Gaussian distribution. To calibrate the variance fluctuation source from protein/peptide intensity differences, a sliding window from low to high intensity was used to select protein/peptide within a specific intensity scale. The maximum likelihood estimation was used to optimize the parameters in Gaussian distribution after removing the top and bottom 5% entities in each sliding window. The significantly changed expression of global proteins was determined by a log_2_ (fold change) consistently above or below 0 plus FDR uniformly lower than 0.1 in all three biological replicates. To capture protein expression pattern changes due to PfNIF4 deficiency, we compared pairwise significantly changed global protein levels between GlcN treated and untreated parasites. We used K-means to further classify proteins/peptides based on their log_2_ (fold change) level to capture the dynamic change through trophozoite to schizont stage (24/36-hpi pair). Akaike information criterion (AIC) and BIC were used to search optimized category numbers of K-means classification. Fisher’s exact test was performed to identify GO or malaria parasite metabolic pathways (MPMP) terms enriched in the specific set. The expected number of proteins in the bar plot indicates the number of proteins with the specific functional term would be observed if the query protein set followed the fraction of protein in the whole genome possessing this function term. The same statistical analysis was conducted for protein domain enrichment and the protein domain information was downloaded from PlasmoDB.

### TurboID and proteomic analysis.

Approximately 300 mL of parasite-iRBC cultures were used to purify biotinylated proteins. After tight synchronization with 40%/70% Percoll purification and 5% sorbitol treatment, schizonts (36 hpi) expressing the TurboID fusion proteins were labeled with biotin (Sangon Biotech, Shanghai, China) at a final concentration of 500 μM at 37°C for 10 min. Labeling was stopped by transferring the mutants to ice and washing them five times with ice-cold PBS. Then the parasite-iRBCs were lysed with 0.15% saponin, and the proteins were extracted using the Pierce IP Lysis Buffer (Thermo Fisher). The extracts were subjected to affinity purification. One mL of biotin-labeled proteins was added to the Streptavidin immunomagnetic beads (Sangon Biotech) and incubated at room temperature for 1 h with mixing. Streptavidin immunomagnetic beads were washed four times with 0.1% Tween 20 in TBS-based solubilization buffer, and 10% of the biotinylated proteins were removed from the magnetic beads with 50 μL of Laemmli SDS-sample buffer saturated with biotin at 98°C. Before mass spectrometry, the eluted proteins were analyzed by Western blotting using an anti-FKBP antibody (Abcam).

For proteomic analysis, protein immunoprecipitation was performed as described (95). Briefly, the proteins bound to streptavidin beads (biotinylated proteins) were directly digested by trypsin. The digested peptides were eluted, dried in a vacuum concentrator, and proceeded for LC-MS/MS analysis as described above. To quantify the proteins present in the affinity purification from the NIF4^TurboID^ parasites but absent from the 3D7 control, we applied the intensity-based LFQ algorithm iBAQ (102, 103). For normalization, the iBAQ values were converted into iFOT by dividing the iBAQ value of each protein by the sum of iBAQ values of all proteins and then multiplied by 10^5^ for easy visualization. The MS raw data (PXD030249) were submitted to the ProteomeXchange Consortium via PRIDE (104).

### Phosphoproteome analysis.

Individual samples prepared from the global proteome analysis were used for phosphoproteome analysis. Phosphopeptide enrichment was carried out using a Ti-IMAC (immobilized metal ion affinity chromatography) microsphere (ReSyn Biosciences, Beijing, China) on the digested proteins for each biological replicate according to the manufacturer’s instruction. The Ti-IMAC microspheres with enriched phosphopeptides were collected by centrifugation at 12,000 × *g* for 5 min. To remove the nonspecific binding, the Ti-IMAC microspheres were washed sequentially with 50% acetonitrile/6% TFA and 30% acetonitrile/0.1% TFA. Phosphopeptides were eluted with 1 mL of 10% NH_4_OH. The supernatant containing phosphopeptides was collected and lyophilized. The following LC-MS/MS analysis was performed as described above. To analyze MS files from the Ti-IMAC enrichment, variable phosphorylation on S, T, and Y was added. The MS proteomic data were deposited to the ProteomeXchange via the PRIDE database (PXD033957).

To determine the relative abundance of the phosphorylated peptides, each phosphorylation intensity value was further normalized to the intensity of the corresponding protein expression (global proteome). An improved Gaussian model was implemented to capture phosphopeptide change after GlcN treatment. The proteins with significant changes in phosphorylation were selected based on the log_2_ (fold change) higher or lower than 0, and adjusted *P* value < 0.1, consistently in all the three biological replicates. The FDR was used to adjust the *P* value. To capture phosphorylation pattern changes due to PfNIF4 KD, a heatmap of K-means classification for the 24/36-hpi pair was performed as described above. GO enrichment analyses were performed as described in proteomic analysis with an FDR-adjusted *P* value lower than 0.05. The featured motifs of phosphorylated peptides were analyzed by using the motif-x algorithm Soft MoMo v5.1.1 (http://meme-suite.org/tools/momo) (105). The query sets of phosphorylated sequences are constituted of 13 (–6, +6) amino acids flanking the phosphorylated amino acid (six amino acids upstream and six amino acids downstream of the phosphorylated site) in all protein sequences. The background or control data sets here are selected according to the type of amino acids phosphorylated in query data. Neighboring (–6, +6) amnio acids around the type of amino acids in query sets are extracted from known proteins in P. falciparum from PlasmoDB. The motif sequence was considered when the minimum number of occurrences was over 20 and the *P* value was <1e − 2.

### Statistical analysis.

Statistical analyses were performed using GraphPad Prism 8.0.1 (GraphPad Software, San Diego, CA, USA). For parasitemia and gametocytemia, data were analyzed by a Student’s *t* test. For comparison of female: male gametocyte ratios and relative expression levels of different genes by RT-PCR at each time point, one-way ANOVA and *t* test were performed with Bonferroni corrections.
