# Supplementary material for: The Plasmodium falciparum Nuclear Protein Phosphatase NIF4 Is Required for Efficient Merozoite Invasion and Regulates Artemisinin Sensitivity
Source: mBio. 2022 Aug 8;13(4):e01897-22. doi: 10.1128/mbio.01897-22 (PMC9426563; doi:10.1128/mbio.01897-22)
Supplement: FIG S1 [file mbio.01897-22-s0001.pdf]

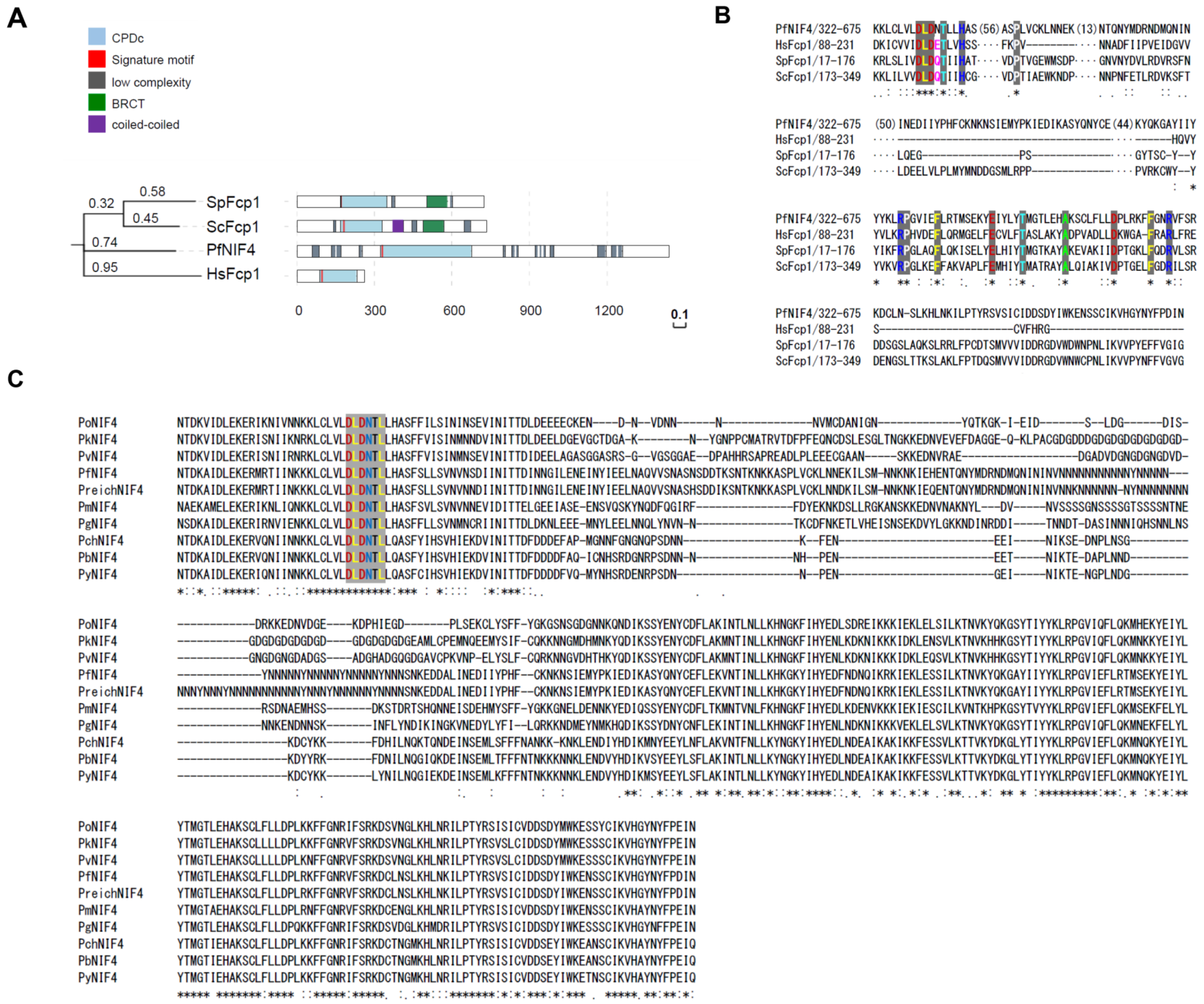
A
CPDc
Signature motif
low complexity
BRCT
coiled-coiled
0.58
0.32
SpFcp1
0.45
ScFcp1
0.74
PfNIF4
0.95
HsFcp1
0
300
600
900
1200
0.1
B
PfNIF4/322-675 KKLCLVLDLDNTLLHAS(56)ASPLVCKLNNEK(13)NTQNYMDRNDMQNIN
HsFcp1/88-231 DKICVVIDLDETLVHSS····FKPV--------····NNADFIIPVEIDGVV
SpFcp1/17-176 KRLSLIVDLDQTIIHAT····VDPTVGEWMSDP····GNVNYDVLRDVRSFN
ScFcp1/173-349 KKLILVVDLDQTIIHCG····VDPTIAEWKNDP····NNPNFETLRDVKSFT
PfNIF4/322-675 (50)INEDIIYPHFCKNKNSIEMYPKIEDIKASYQNYCE(44)KYQKGAYIIY
HsFcp1/88-231 ····-----------------------------····-----HQVY
SpFcp1/17-176 ····LQEG----------PS----------····GYTSC-Y--Y
ScFcp1/173-349 ····LDEELVLPLMYMNDDGSMLRPP----------····PVRKCWY--Y
PfNIF4/322-675 YYKLRPGVIEFLRTMSEKYEIYLYTMGTLEHAKSCLFLLDPLRKFFGNRVFSR
HsFcp1/88-231 YVLKRPHVDEFLQRMGELFECVLFTASLAKYADPVADLLDKWGA-FRARLFRE
SpFcp1/17-176 YIKFRPGLAQFLQKISELYELHIYTMGTKAYAKEVAKIIDPTGKLFQDRVLSR
ScFcp1/173-349 YVKVRPGLKEFFAKVAPLFEMHIYTMATRAYALQIAKIVDPTGELFGDRILSR
PfNIF4/322-675 KDCLN-SLKHLNKILPTYRSVSICIDDSDYIWKENSSCIKVHGYNYFPDIN
HsFcp1/88-231 S--------------------CVFHRG--------------------
SpFcp1/17-176 DDSGSLAQKSLRRLFPCDTSMVVVIDDRGDVWDWNPNLIKVVPYEFFVGIG
ScFcp1/173-349 DENGSLTTKSLAKLFPTDQSMVVVIDDRGDVWNWCPNLIKVVPYNFFVGVG
C
PoNIF4 NTDKVIDLEKERIKNIVNNKKLCLVLDLDNTLLHASFFILSININSEVINITTDLDEEEECKEN----D-N--VDNN------N-------------NVMCDANIGN------------YQTKGK-I-EID-------S--LDG------DIS-
PkNIF4 NTDKVIDLEKERISNIIKNRKLCLVLDLDNTLLHASFFVISINMNNDVINITTDLDEELDGEVGCTDGA-K--------N--YGNPPCMATRVTDFPFEQNCDSLESGLTNGKKEDNVEVEFDAGGE-Q-KLPACGDGDDDGDGDGDGDGDGDGD-
PvNIF4 NTDKVIDLEKERISNIIRNRKLCLVLDLDNTLLHASFFVISINMNSEVINITTDIDEELAGASGGASRS-G--VGSGGAE--DPAHHRSAPREADLPLEEECGAAN-----SKKEDNVRAE-----------------DGADVDGNGDGNGDVD-
PfNIF4 NTDKAIDLEKERMRTIINKKKLCLVLDLDNTLLHASFSLLSVNVNSDIINITTDINNGILENEINYIEELNAQVVSNASNSDDTKSNTKNKKASPLVCKLNNEKILSM-NNKNKIEHENTQNYMDRNDMQNININVNNNNNNNNNNYNNNNN---
PreichNIF4 NTDKAIDLEKERMRTIINKKKLCLVLDLDNTLLHASFSLLSVNVNNDIINITTDINNGILENEINYIEELNAQVVSNASHSDDIKSNTKNKKASPLVCKLNNDKILSM-NNKNKIEQENTQNYMDRNDMQNININVNNKNNNNN-NYNNNNNNNN
PmNIF4 NAEKAMELEKERIKNLIQNKKLCLVLDLDNTLLHASFSVLSVNVNNEVIDITTELGEEIASE-ENSVQSKYNQDFQGIRF--------------FDYEKNKDSLLRGKANSKKEDNVNAKNYL---DV-----NVSSSSGNSSSSGTSSSSNTNE
PgNIF4 NSDKAIDLEKERIRNVIENKKLCLVLDLDNTLLHASFFLLSVNMNCRIINITTDLDKNLEEE-MNYLEELNNQLYNVN-N--------------TKCDFNKETLVHEISNSEKDVYLGKKNDINRDDI-----TNNDT-DASINNNIQHSNNLNS
PchNIF4 NTDKAIDLEKERVQNIINNKKLCLVLDLDNTLQASFYIHSVHIEKDVINITTDFDDDEFAP-MGNNFGNGNQPSDNN-----------------K--FEN----------------------EEI-----NIKSE-DNPLNSG---------
PbNIF4 NTDKAIDLEKERVQNIINNKKLCLVLDLDNTLQASFYIHSVHIEKDVINITTDFDDDDFAQ-ICNHSRDGNRPSDNN-N--------------NH--PEN----------------------EET-----NIKTE-DAPLNND---------
PyNIF4 NTDKAIDLEKERIQNIINNKKLCLVLDLDNTLQASFCIHSVHIEKDVINITTDFDDDDFVQ-MYNHSRDENRPSDN-----------------N--PEN----------------------GEI-----NIKTE-NGPLNDG---------
PoNIF4 ------------DRKKEDNVDGE----KDPHIEGD-------PLSEKCLYSFF-YGKGSNSGDGNNKQNDIKSSYENYCDFLAKINTLNLLKHNGKFIHYEDLSDREIKKKIEKLELSILKTNVKYQKGSYTIYYKLRPGVIQFLQKMHEKYEIYL
PkNIF4 -----------GDGDGDGDGDGD----GDGDGDGDGEAMLCPEMNQEEMYSIF-CQKKNNGMDHMNKYQDIKSSYENYCDFLAKMNTINLLKHNGKFIHYENLKDKNIKKKIDKLENSVLKTNVKHHKGSYTIYYKLRPGVIQFLQKMNKKYEIYL
PvNIF4 -----------GNGDGNGDADGS----ADGHADGQGDGAVCPKVNP-ELYSLF-CQRKNNGVDHTHKYQDIKSSYENYCDFLAKMNTINLLKHNGKFIHYENLKDKNIKKKIEQSVLKTNVKHHKGSYTIYYKLRPGVIQFLQKMNKKYEIYL
PfNIF4 -------------YNNNNNYNNNNNYNNNNNYNNNSNKEDDALINEDIIYPHF--CKNKNSIEMYPKIEDIKASYQNYCEFLEKVNTINLLKHKGKYIHYEDFNDNQIKRKIEKLESSILKTNVKYQKGAYIIYYKLRPGVIEFLRTMSEKYEIYL
PreichNIF4 NNNYNNNYNNNNNNNNNNNYNNNYNNNNNNYNNNNSNKEDDALINEDIIYPHF--CKNKNSIEMYPKIEDIKASYQNYCEFLEKVNTINLLKHKGKYIHYEDFNDNQIKRKIEKLESSILKTNVKYQKGAYIIYYKLRPGVIEFLRTMSEKYEIYL
PmNIF4 -------------RSDNAEMHSS-------DKSTDRTSHQNNEISDEHMYSFF-YGKKGNELDENNKYEDIQSSYENYCDFLTKMNTVNLFKHNGKFIHYEDLKDENVKKKIEKIESCILKVNTKHPKGSYTVYYKLRPGVIEFLQKMSEKFELYL
PgNIF4 -------------NNKENDNNSK-------INFLYNDIKINGKVNEDYLYFI--LQRKKNDMEYNMKHQDIKSSYDNYCNFLEKINTINLLKHNGKFIHYENLNDKNIKKKVEKLELSVLKTNVKYQKGSYTIYYKLRPGVIQFLQKMNEKFELYL
PchNIF4 -----------------KDCYKK-------FDHILNQKTQNDEINSEMLSFFFNANKK-KNKLENDIYHDIKMNYEEYLNFLAKVNTFNLLKYNGKYIHYEDLNDEAIKAKIKKFESSVLKTTVKYDKGLYTIYYKLRPGVIEFLQKMNQKYEIYL
PbNIF4 -----------------KDYYRK-------FDNILNQGIQKDEINSEMLTFFFNTNKKKNNKLENDVYHDIKVSYEEYLSFLAKINTLNLLKYNGKYIHYEDLNDEAIKAKIKKFESSVLKTTVKYDKGLYTIYYKLRPGVIEFLQKMNQKYEIYL
PyNIF4 -----------------KDCYKK-------LYNILNQGIEKDEINSEMLKFFFNTNKKKNNKLENDVYHDIKMSYEEYLSFLAKINTLNLLKYNGKYIHYEDLNDEAIKAKIKKFESSVLKTTVKYDKGLYTIYYKLRPGVIEFLQKMNQKYEIYL
PoNIF4 YTMGTLEHAKSCLFLLDPLKKFFGNRIFSRKDSVNGLKHLNRILPTYRSISICVDDSDYMWKESSYCIKVHGYNYFPEIN
PkNIF4 YTMGTLEHAKSCLLLLDPLKKFFGNRVFSRKDSVNGLKHLNRILPTYRSVSLCIDDSDYMWKESSSCIKVHGYNYFPEIN
PvNIF4 YTMGTLEHAKSCLLLLDPLKNFFGNRVFSRKDSVNGLKHLNRILPTYRSVSLCIDDSDYMWKESSSCIKVHGYNYFPEIN
PfNIF4 YTMGTLEHAKSCLFLLDPLRKFFGNRVFSRKDCLNSLKHLNKILPTYRSVSICIDDSDYIWKENSSCIKVHGYNYFPDIN
PreichNIF4 YTMGTLEHAKSCLFLLDPLRKFFGNRVFSRKDCLNSLKHLNKILPTYRSVSICIDDSDYIWKENSSCIKVHGYNYFPDIN
PmNIF4 YTMGTAEHAKSCLFLLDPLRNFFGNRVFSRKDCENGLKHLNRILPTYRSISICVDDSDYIWKENSSCIKVHAYNYFPEIN
PgNIF4 YTMGTLEHAKSCLFLLDPQKKFFGNRIFSRKDSVDGLKHMDRILPTYRSVSICIDDSDYIWKESSSCIKVHGYNFFPEIN
PchNIF4 YTMGTIEHAKSCLFLLDPLKKFFGNRIFSRKDCTNGMKHLNRILPTYRSISICVDDSEYIWKEANSCIKVHAYNYFPEIQ
PbNIF4 YTMGTIEHAKSCLFLLDPLKKFFGNRIFSRKDCTNGMKHLNRILPTYRSISICVDDSEYIWKEANSCIKVHAYNYFPEIQ
PyNIF4 YTMGTIEHAKSCLFLLDPLKKFFGNRIFSRKDCTNGMKHLNRILPTYRSISICVDDSEYIWKETNSCIKVHAYNYFPEIQ

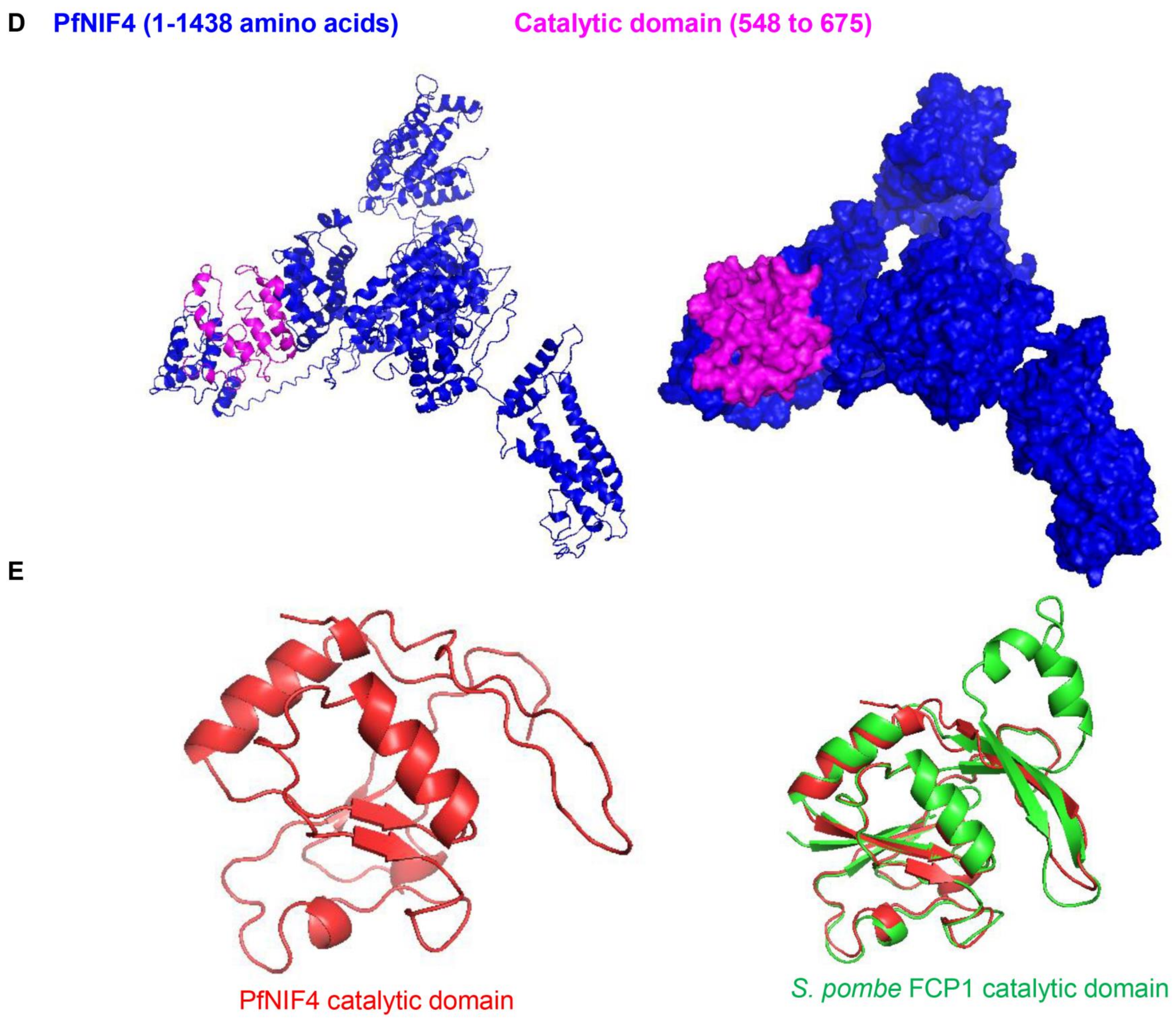
D
PfNIF4 (1-1438 amino acids)
Catalytic domain (548 to 675)
E
PfNIF4 catalytic domain
S. pombe FCP1 catalytic domain

FIG S1. Bioinformatics of NIF4 protein in *P. falciparum*. (**A)** Phylogenetic analysis of NIF4 orthologues in *Plasmodium falciparum* NIF4 (PfNIF4), Saccharomyces cerevisiae Fcp1 (ScFcp1), *Schizosaccharomyces pombe* Fcp1 (SpFcp1), and human Scp1 (HsScp1). Tree shown is the consensus of trees from four independent inferences based on a trimmed alignment of the full length of proteins. Schematic representations of the predicted protein architectures (Pfam domains) of the NIF4 orthologues are shown in right panel. Domain organization of *Plasmodium falciparum* NIF4 (PfNIF4), *Saccharomyces cerevisiae* Fcp1 (ScFcp1), *Schizosaccharomyces pombe* Fcp1 (SpFcp1), and human Scp1 (HsScp1). CPDc and BRCT denote the catalytic domain of ctd-like phosphatases regions and the breast cancer protein-related carboxy-terminal domains, respectively. The signature motif, DxDx(T/V) are depicted with red lines. The bootstrap values are indicated on each branch. **(B)** Alignment of PfNIF4, SpFcp1, ScFcp1, and HsScp1 with the CPDc domains of Fcp1 from *Plasmodium falciparum*, *Schizosaccharomyces pombe* (Sp), and *Saccharomyces cerevisiae* (Sc) and human (Hs). Conserved residues are marked with an asterisk below the alignment. **(C)** Sequence alignment of CPDc regions from NIF4 orthologues in *Plasmodium spp*. Sequences of the CPDcs drawn from NCBI: *P. ovale*: PocGH01_08021100; *P. knowlesi* Strain H: PKNH_0812500; *P. vivax* Sal-1: PVX_094855; *P. falciparum* 3D7: PF3D7_1012700; *P. reichinowi*: PRCDC_1012100; *P. malariae*: PmUG01_08028700; *P. gallinaceum*: PGAL8A_00388900; *P. chabaudi*: PCHAS_1211800; *P. berghei*: PBANKA_1211100; *P. yoelii*17XNL: PY03480. The CPDc regions are marked with blue lines. The signature motif, DLDNT are depicted with black arrows. **(D)** The 3-D structure of PfNIF4 predcited by I-TASSER with the catalytic domain shown in pink color. Left, ribbon diagram; right, space-filling model. **(E)** Predicted structure of the ctd-like phosphatase catalytic domain of PfNIF4 (left) and superimposition of the PfNIF4 catalytic domain (red) with the FCP1 (green) from *Schizosaccharomyces pombe*.
