# Supplementary material for: The Plasmodium falciparum Nuclear Protein Phosphatase NIF4 Is Required for Efficient Merozoite Invasion and Regulates Artemisinin Sensitivity
Source: mBio. 2022 Aug 8;13(4):e01897-22. doi: 10.1128/mbio.01897-22 (PMC9426563; doi:10.1128/mbio.01897-22)
Supplement: FIG S2 [file mbio.01897-22-s0002.pdf]

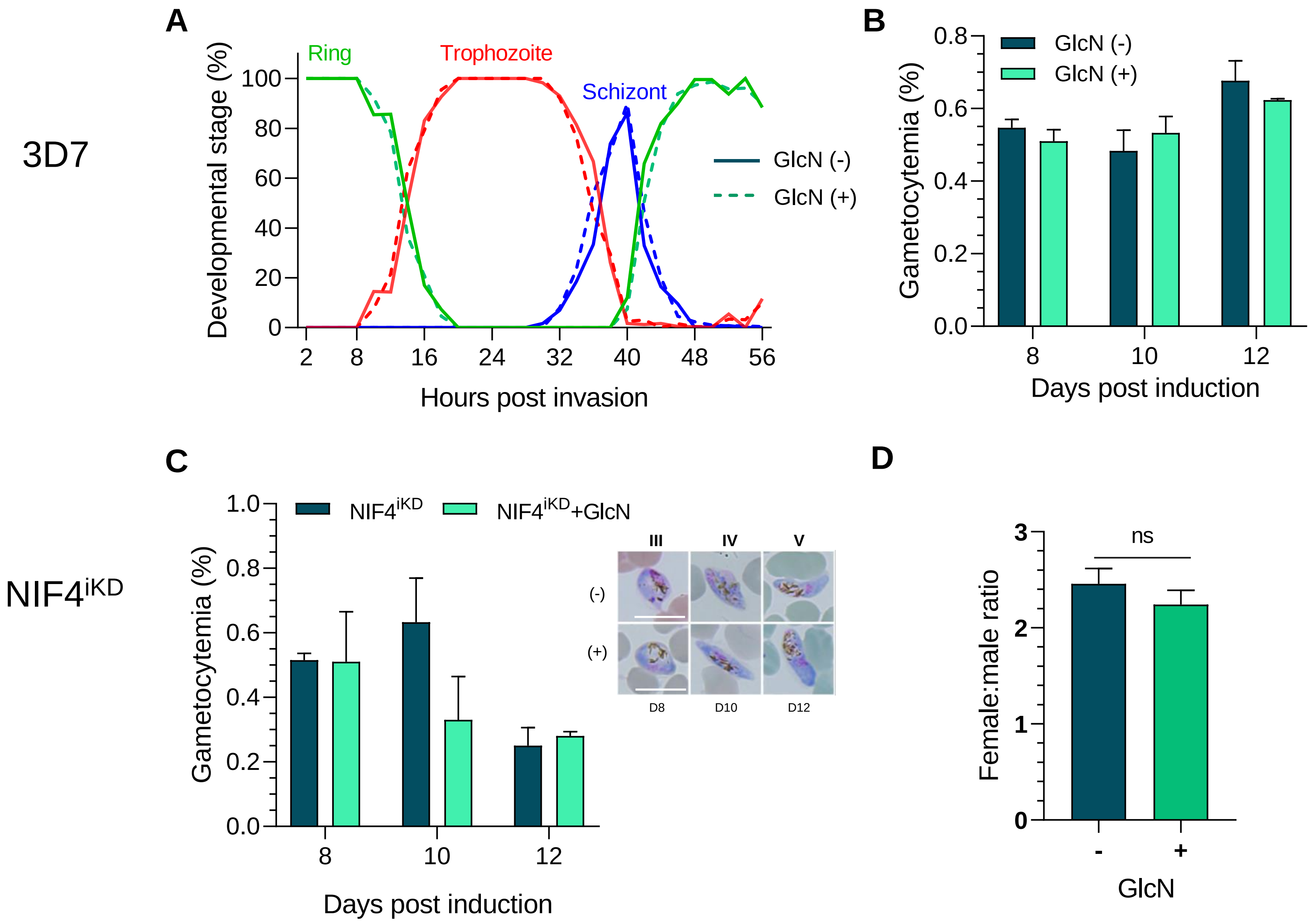

FIG S2. The effect of 2.5 mM GlcN on wild-type 3D7 (A, B) and NIF4^{iKD} (C, D) parasites. (A) The intraerythrocytic cycle of 3D7 parasites in the absence (solid lines) or presence (dashed lines) of 2.5 mM GlcN. **(B)** Gametocytemia of 3D7 parasites in the presence or absence of 2.5 mM GlcN. **(C)** Gametocytemia of the NIF4^{iKD} parasites (C3 clone) in the presence or absence of 2.5 mM GlcN. Representatives of Giemsa-stained gametocytes from stage III to V were shown on the right panel. Scale bars, 10 µm. **(D)** The female:male ratio of mature gametocytes. Mature male and female gametocytes were differentiated based on morphological differences of Giemsa-stained gametocytes. For each time point in panels B, C, D after induction of gametocytogenesis, the data represent the mean ± SEM of three biological replicates.
