# Supplementary material for: The Plasmodium falciparum Nuclear Protein Phosphatase NIF4 Is Required for Efficient Merozoite Invasion and Regulates Artemisinin Sensitivity
Source: mBio. 2022 Aug 8;13(4):e01897-22. doi: 10.1128/mbio.01897-22 (PMC9426563; doi:10.1128/mbio.01897-22)
Supplement: FIG S3 [file mbio.01897-22-s0003.pdf]

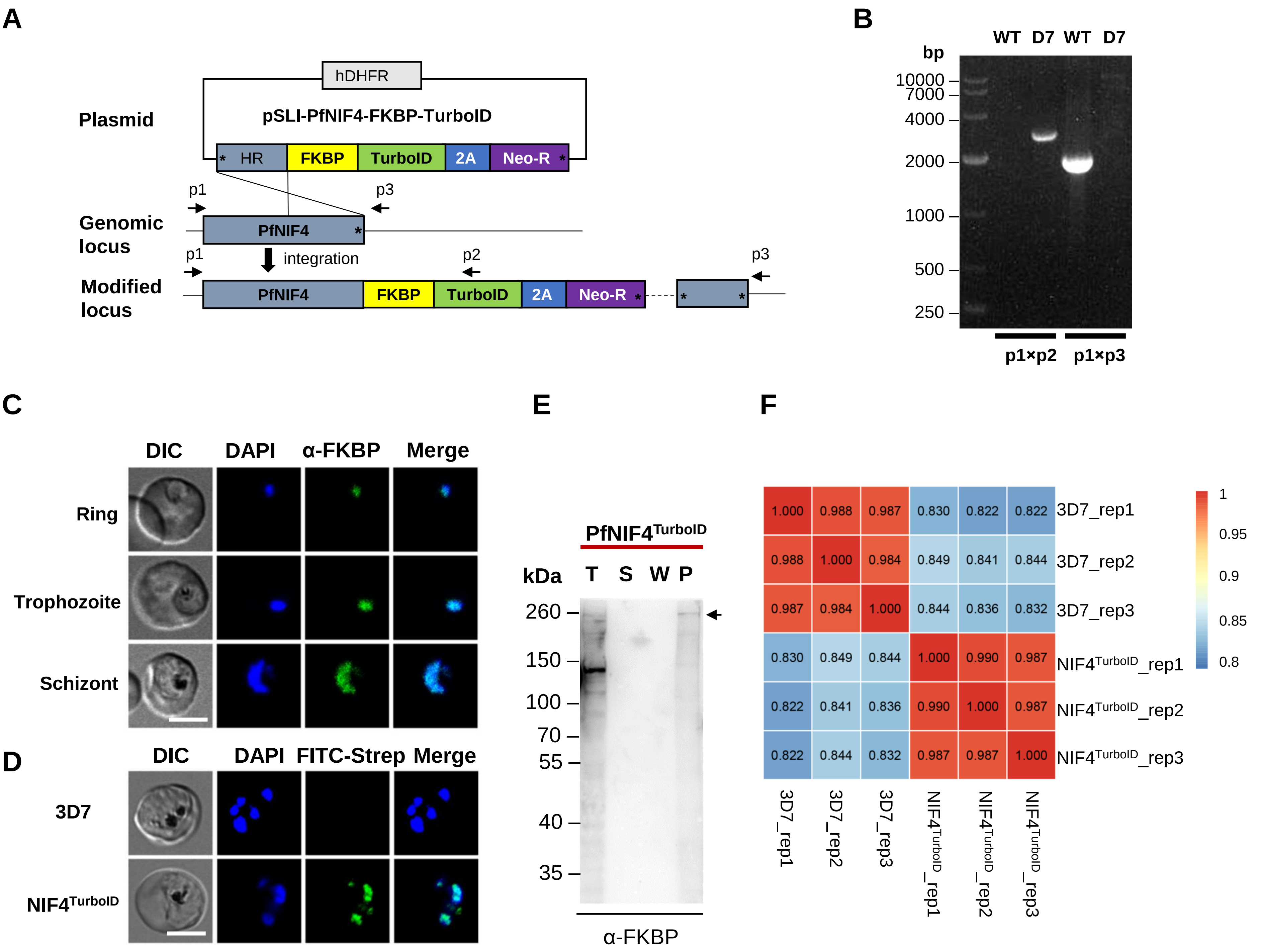

	3D7_rep1	3D7_rep2	3D7_rep3	NIF4TurboID_rep1	NIF4TurboID_rep2	NIF4TurboID_rep3
3D7_rep1	1.000	0.988	0.987	0.830	0.822	0.822
3D7_rep2	0.988	1.000	0.984	0.849	0.841	0.844
3D7_rep3	0.987	0.984	1.000	0.844	0.836	0.832
NIF4TurboID_rep1	0.830	0.849	0.844	1.000	0.990	0.987
NIF4TurboID_rep2	0.822	0.841	0.836	0.990	1.000	0.987
NIF4TurboID_rep3	0.822	0.844	0.832	0.987	0.987	1.000

FIG S3. Tagging of PfNIF4 with the TurboID and mass spectrometric analysis. (A) Schematic representation of the SLI-based single-crossover homologous recombination approach resulting in a PfNIF4-FKBP-turboID fusion at the endogenous locus. FKBP, TurboID ligase, and the skip peptide 2A sequence are shown as yellow, green, and blue boxes, respectively. The hDHFR (human dihydrofolate reductase) and Neo-R (neomycin resistance) boxes indicate the two drug selection cassettes. HR, homology region for recombination; *, stop codons; p1, p2, and p3, primers used for verifying integration by PCR. **(B)** PCR analysis with the primer pairs p1×p2 and p1×p3 using genomic DNA from 3D7(WT) and PfNIF4^{TurboID} (clone D7) parasites. DNA ladder size indicated in base pairs (bp). Correct integration into the endogenous locus was detected by primers p1×p2 (2698 bp), while the intact endogenous locus was detected by primers p1×p3 (1901 bp). **(C)** Localization of PfNIF4-FKBP-TurboID to the nucleus in asexual blood stages. Representative IFA images of PfNIF4-FKBP-TurboID expression in NIF4^{TurboID} parasites at the ring, trophozoite, and schizont stages, detected using the anti-FKBP antibody. Nuclei were stained with DAPI. DIC, differential interference contrast. Scale bar, 5 µm. **(D)** Nuclear localization of biotinylated proteins in the NIF4^{TurboID} and 3D7 control parasites. Schizonts were incubated with 500 µM biotin for 10 min and visualized by FITC-conjugated streptavidin (green). Nuclei were stained with DAPI (blue). Scale bar, 5 µm. **(E)** Western blot analysis of biotin-labeled samples precipitated using streptavidin magnetic beads. The blot was probed with the anti-FKBP antibody. T, total input protein; S, supernatant; W, wash; P, pellet. Arrow indicates the recombinant PfNIF4-FKBP-TurboID fusion protein with a molecular size of ~237 kDa. **(F)** Hierarchical clustering of pairwise Pearson correlation coefficients of the proteomic data of precipitated proteins from three biological replicates.
