# Supplementary material for: The Plasmodium falciparum Nuclear Protein Phosphatase NIF4 Is Required for Efficient Merozoite Invasion and Regulates Artemisinin Sensitivity
Source: mBio. 2022 Aug 8;13(4):e01897-22. doi: 10.1128/mbio.01897-22 (PMC9426563; doi:10.1128/mbio.01897-22)
Supplement: FIG S4 [file mbio.01897-22-s0004.pdf]

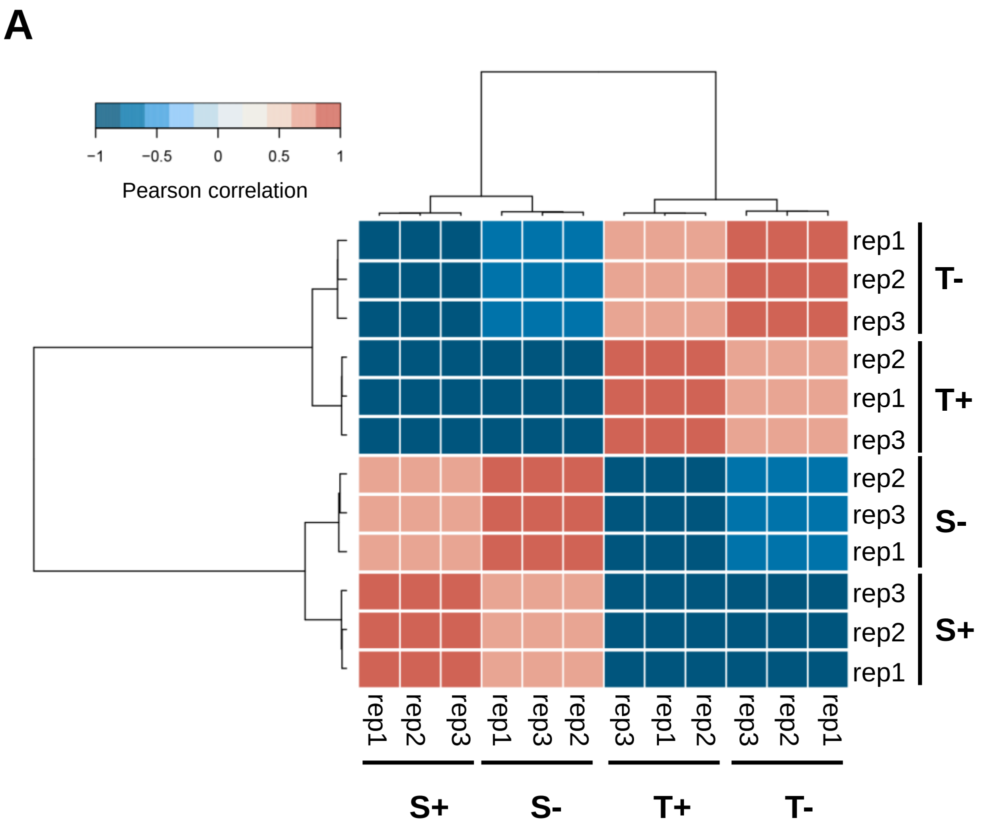

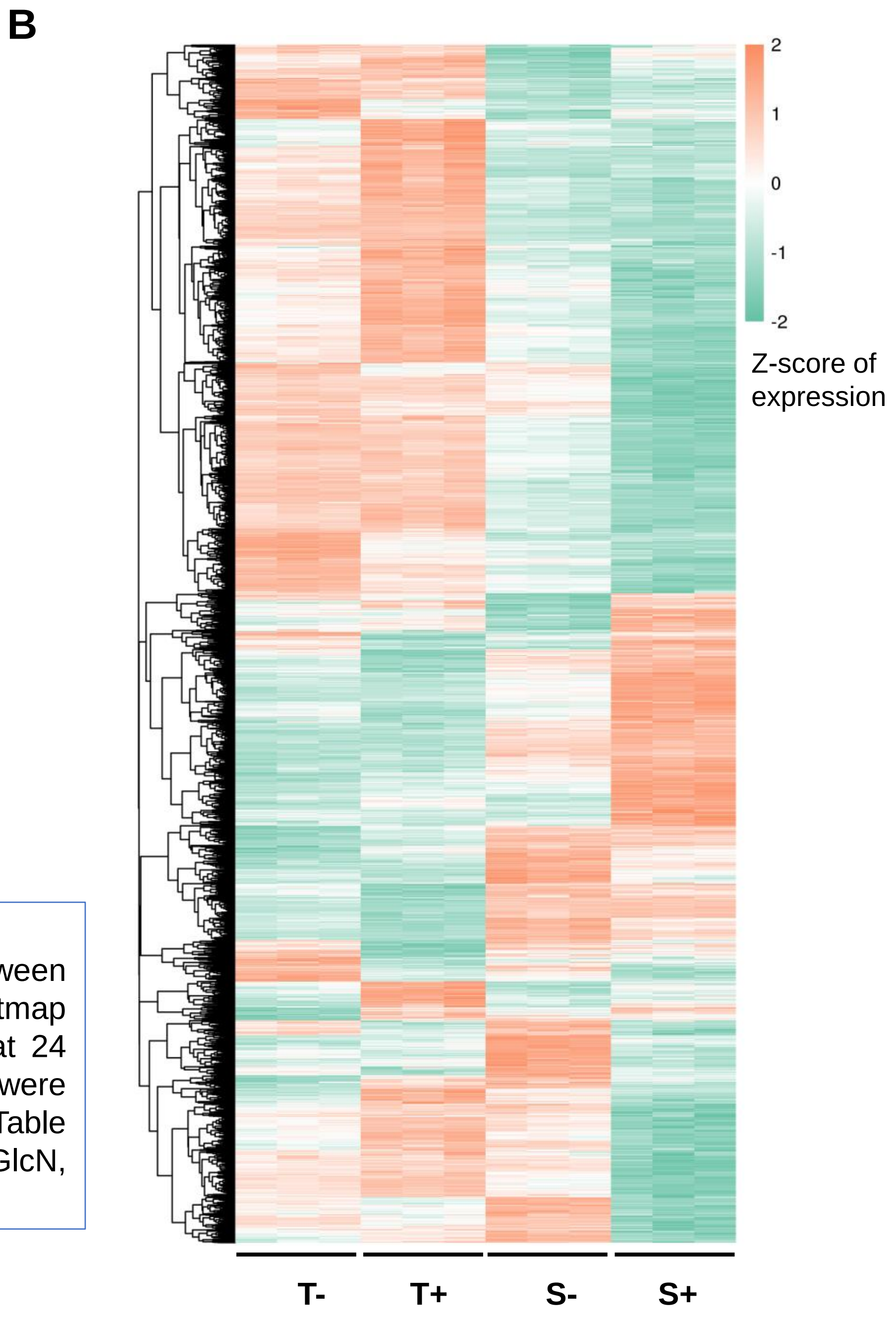

FIG S4. Phosphoproteomic profiling of NIF4^{iKD} parasites.
(A) Hierarchical clustering of Pearson correlation coefficients between each pair of samples from three biological replicates. **(B)** Heatmap showing changes in phosphosite levels upon PfNIF4 knockdown at 24 and 36 hpi. Phosphosite levels (Supplementary Data table S5) were normalized based on scaled protein levels (Supplementary Data Table S3). T, trophozoites; S, schizonts. + and – denote with and without GlcN, respectively.
