# Supplementary material for: The Plasmodium falciparum Nuclear Protein Phosphatase NIF4 Is Required for Efficient Merozoite Invasion and Regulates Artemisinin Sensitivity
Source: mBio. 2022 Aug 8;13(4):e01897-22. doi: 10.1128/mbio.01897-22 (PMC9426563; doi:10.1128/mbio.01897-22)
Supplement: FIG S5 [file mbio.01897-22-s0005.pdf]

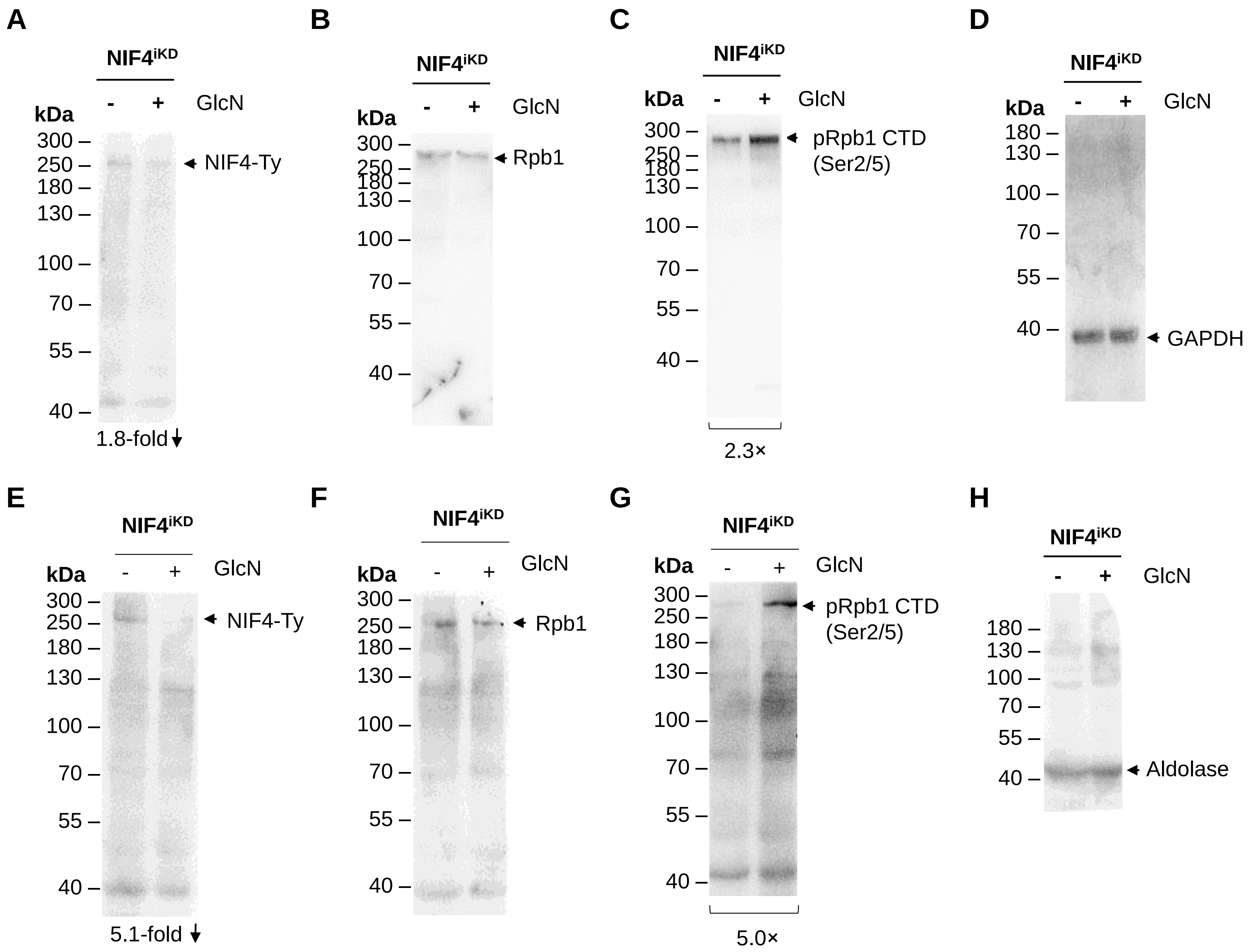

FIG S5. Raw Western blot data related to FIG 7D. Arrows indicate bands relevant to the detected protein. A-D, first cycle; E-H, second cycle. Molecular weight markers are shown in kDa.
