# Supplementary material for: The Plasmodium falciparum Nuclear Protein Phosphatase NIF4 Is Required for Efficient Merozoite Invasion and Regulates Artemisinin Sensitivity
Source: mBio. 2022 Aug 8;13(4):e01897-22. doi: 10.1128/mbio.01897-22 (PMC9426563; doi:10.1128/mbio.01897-22)
Supplement: TABLE S1 [file mbio.01897-22-s0006.docx]

Table S1. Primers used in this study

| primers | sequence (5’ to 3’) |
| --- | --- |
| NIF4^iKD^-AsciF1 | gcggccctagtctagGGcGCGCCCCTGTTGTGCGTACGATATTGAC |
| NIF4^iKD^-R1 | gctagcTTTTTTATTTTCTTCATATTCAATGACATCCC |
| NIF4^iKD^-F | GCATGCataatatgttatctcttacac |
| NIF4^iKD^-AflII1 | tttttttacaaaatgcttaatCACATTTTTTATGAACTGTGTGG |
| NIF4^iKD^-SgRNAF1 | taagtatataatattTTTATTACTTCCAAGTATAAAGGgttttagagctagaa |
| NIF4^iKD^-SgRNAR1 | ttctagctctaaaacCCTTTATACTTGGAAGTAATAAAaatattatatactta |
| 3Ty+glmS.F | aataagaaaataatgGCTAGCGGCTCTGGCAGCGGCAGCG |
| 3Ty+glmS.R | atatgtgcgtgtgagGCATGCGTCCCCTCCTACATGTTTTTTGG |
| NIF4^iKD^-genoF1 | CAAATGACGAAAATAGAGATG |
| NIF4^iKD^-genoR1 | CCCTCTATAATGTTCATACATAC |
| NIF4^iKD^-genoR2 | GTCACCCCCTTGGTTTGAAG |
| pSLI-2xFKBP-GFP-turboID.F1 | gaggtgcaggtagacgtacgATGGGCAAGCCCATCCCCAACCCC |
| pSLI-2xFKBP-GFP-turboID.R1 | cctcttccttctccgtcgacGTCCAGGGTCAGGCGCTCCAG |
| NIF4-Cter.F | tatagaatactcgcggccgctaaGTTCAAAACATGTGAACGAAGTC |
| NIF4-Cter.R | GATCTCAATCCTGAcctaggTTTTTTATTTTCTTCATATTCAATGACATCC |
| pSLI-NIF4-V5-turboID-genoF1 | CCTCTCAAGTGATTGAAGAGGAAC |
| TurboID-R1 | CCATTTGACTCGCACCTTGTC |
| pSLI-NIF4-V5-turboID-genoR2 | CTTAAAGGACACCTTTATATAATTAC |
| PF3D7_1452000.rtF1 | GTTAAAATTTTTCATATTCATTTTAC |
| PF3D7_1452000.rtR1 | CAGGATATTTAGTAGTAGGATATTC |
| PF3D7_1252100.rtF1 | GAATAAATATTGGTTGTATATTG |
| PF3D7_1252100.rtR1 | GGCAACATAGTACTGGTTAAATATTG |
| PF3D7_1116000.rtF1 | GTCTAGTGTTAGATTTTTTTTATG |
| PF3D7_1116000.rtR1 | GTTGATTATTAGTTATATTTTCTTG |
| PF3D7_0817700.rtF1 | GTTGAAATACACTTTGCTC |
| PF3D7_0817700.rtR1 | CTTGTCCTAGAAGGGACGAACC |
| PF3D7_0214900.rtF1 | GCAATATTTTTTTCTGGTATTTC |
| PF3D7_0214900.rtR1 | GCTAGTATGTGTTCCATAAG |
| PF3D7_1017100.rtF1 | GAAAAGAGTATATACGTGTTTG |
| PF3D7_1017100.rtR1 | GTTTTTCATACATTTCTTTATTC |
| PF3D7_1133400.rtF1 | CTGCGTATTATTATTGAGCGCC |
| PF3D7_1133400.rtR1 | GCGTGTTGTAATGTATTTTCG |
| PF3D7_0929400.rtF1 | GATAAAAGTGACAATATTTTTG |
| PF3D7_0929400.rtR1 | CTTATGTTCCCAATAACATTGTG |
| PF3D7_0905400.rtF1 | GCGTAGTAAGCATTTAGTAAC |
| PF3D7_0905400.rtR1 | GTTCCACGAACCAGTAAAATTG |
| PF3D7_0206800.rtF1 | GAAAGTAAATATAGCAACAC |
| PF3D7_0206800.rtR1 | GTTGTGGTAGTTGTGGTAGTTTTG |
| PF3D7_1035400.rtF1 | GAAAAGTTTTATAAATATTAC |
| PF3D7_1035400.rtR1 | CTTTTAATTCTTCCGTATAACC |
| PF3D7_1014100.rtF1 | GTTTTGGTTATCTCTTGTATC |
| PF3D7_1014100.rtR1 | CGAATCCTTTGGATATAATTC |
| PF3D7_0731500.rtF1 | GCTTCCTTCTTTGTGTTATATTTTG |
| PF3D7_0731500.rtR1 | GATGGAAGAGTTATGGAACTCCAG |
